# Dimeric Thymosin β4 Loaded Nanofibrous Interface Enhanced Regeneration of Muscular Artery in Aging Body through Modulating Perivascular Adipose Stem Cell–Macrophage Interaction

**DOI:** 10.1002/advs.201903307

**Published:** 2020-03-16

**Authors:** Wanli Chen, Sansan Jia, Xinchi Zhang, Siqian Zhang, Huan Liu, Xin Yang, Cun Zhang, Wei Wu

**Affiliations:** ^1^ State Key Laboratory of Military Stomatology & National Clinical Research Center for Oral Diseases & Shaanxi Key Laboratory of Stomatology Department of Oral & Maxillofacial Surgery School of Stomatology the Fourth Military Medical University Xi'an Shaanxi China; ^2^ Department of Pathophysiology Institute of Basic Medical Science Xi'an Medical University Xi'an China; ^3^ State Key Laboratory of Cancer Biology Biotechnology Center School of Pharmacy the Fourth Military Medical University Xi'an China

**Keywords:** aging, arterial regeneration, biodegradable grafts, dimeric thymosin‐β4, M2 macrophages

## Abstract

Regenerating nonthrombotic and compliant artery, especially in the aging body, remains a major surgical challenge, mainly owing to the inadequate knowledge of the major cell sources contributing to arterial regeneration and insufficient bioactivity of delivered peptides in grafts. Ultrathin nanofibrous sheaths stented with biodegrading elastomer present opening channels and reduced material residue, enabling fast cell recruitment and host remodeling, while incorporating peptides offering developmental cues are challenging. In this study, a recombinant human thymosin β4 dimer (DTβ4) that contains two complete Tβ4 molecules is produced. The adult perivascular adipose is found as the dominant source of vascular progenitors which, when stimulated by the DTβ4‐loaded nanofibrous sheath, enables 100% patency rates, near‐complete structural as well as adequate functional regeneration of artery, and effectively ameliorates aging‐induced defective regeneration. As compared with Tβ4, DTβ4 exhibits durable regenerative activity including recruiting more progenitors for endothelial cells and smooth muscle cells, when incorporated into the ultrathin polycaprolactone sheath. Moreover, the DTβ4‐loaded interface promotes smooth muscle cells differentiation, mainly through promoting M2 macrophage polarization and chemokines. Incorporating artificial DTβ4 into ultrathin sheaths of fast degrading vascular grafts creates an effective interface for sufficient muscular remodeling thus offering a robust tool for vessel replacement.

## Graphical abstract

Adult perivascular adipose tissue (PVAT) is the dominant source of vascular progenitors in a rat abdominal aorta model. Furthermore, the mechanism by which DTβ4 enhances arterial muscle remodeling in the aging body is that the DTβ4‐incorporated nanofibrous interface facilitates macrophage polarization to the M2 subset, which in turn promotes myogenic differentiation of PVAT‐derived stem cells through secreting platelet‐derived growth factor‐BB (PDGF‐BB).

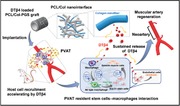

## 1 Introduction

Arterial wall possesses the unique ability to recover endothelium and media following mild vessel injury or implanting nonthrombogenic vascular grafts.^[^ ^1^, ^2^ ^]^ This regenerative capacity mainly attributed to resident stem cells known as vascular progenitor cells (VPCs), which presents a wide spectrum of differentiation potential, including smooth muscle cells (SMCs), pericyte‐like cells, and CD34+ angiogenic progenitor cells.^[^ ^3^, ^4^, ^5^ ^]^ Although there are currently no established markers, quiescent VPCs express Sca‐1,^[^ ^6^, ^7^ ^]^ and lineage tracing determined that most of these cells probably constitutively resident within the vascular wall, especially in adventitia layer.^[^ ^8^ ^]^ During vessel repair, VPCs become activated and generated pools of proliferative myogenic precursor cells capable of rebuilding muscular wall.^[^ ^9^, ^10^ ^]^ Regulating the activity of VPCs are orchestrated waves of pro‐ and antiinflammatory factors primarily released from infiltrating macrophages helping to stimulate myogenic activation and differentiation.^[^ ^11^ ^]^ Although normally robust, the regeneration process can be impaired in cases of aging, chronic degenerative diseases, inappropriate grafting materials resulting in environments that impaired regenerative capacity of VPCs.^[^ ^12^, ^13^ ^]^

Recent advances in tissue‐engineered blood vessels have enabled the modeling and improved understanding of complex biological phenomena.^[^ ^14^, ^15^ ^]^ Specifically, various methods to recreate critical components of the muscular remodeling have been developed to systematically study the cellular events and interactions involved in VPCs. Incorporating chemokines promoting stem cells recruitment in polymeric grafts and young cells engineered extracellular matrices have proven capable in promoting VPCs homing and myogenic differentiation.^[^ ^2^, ^16^ ^]^ In such biomimetic systems, the interactions between the VPCs and developmental signals have been replicated. Modification of synthetic grafts aims at promoting homing of progenitor cells by delivering chemotaxis factors or stem cell activators have been pursued as potential means to regenerate functional artery.^[^ ^17^, ^18^ ^]^ However, the critical roles of the immune cells widely playing roles in adult tissues repair, especially during biomaterial mediated regeneration, are scarcely considered.

Utilizing fast‐degrading polymer, such as poly‐glycerol sebacate (PGS), as vascular grafts would greatly reduce material residue thus ameliorates chronic inflammation. The ultrathin polycaprolactone (PCL) nanofiber sheath around PGS tubes allowed transmural cell migration thus enabled fast muscular remodeling.^[^ ^19^ ^]^ However, opening and fast degrading properties encountered catastrophic risks caused by mechanical loss, such as aneurysmal dilatation or graft rupture, especially in the context of clinical application for patients with impaired progenitor function.^[^ ^20^ ^]^ Increasing nanofiber density and thickness are routinely applied to enhance mechanical strength of PCL sheath, whereas greatly influenced the remodeling results varying from functional tissues to fibrotic scar, mainly through activating proinflammatory macrophages and reducing progenitor cells recruitment.^[^ ^21^, ^22^ ^]^ Optimizing components of nanofibrous interface, so that inflammatory cells‐VPCs interaction could be modulated to favor the muscular remodeling would be critical for further clinical translation. However, two major issues remain to be resolved: i) inadequate knowledge on major cell source contributing to arterial regeneration specific to fast degrading grafts and ii) insufficient bioactivity of the delivered protein in nanofibrous sheath.

Thymosin β4 (Tβ4), a thymic hormone with multiple intracellular and extracellular activities, is known for its role in regulating cardiac vessel formation (vasculogenesis, angiogenesis, and arteriogenesis).^[^ ^23^, ^24^ ^]^ Recently, it is revealed that Tβ4 exerts both antiinflammatory and antifibrotic effects during tissue healing, notably following alkali eye injury and blebomyocin‐induced damage in lung.^[^ ^25^ ^]^ It was further found that Tβ4 promotes inflammation resolution in chronic granulomatous disease, suggesting participation of Tβ4 in modulating innate immunity.^[^ ^26^ ^]^ Reducing inflammation is pivotal in preventing biomaterial‐induced fibrosis and functional regeneration. Despite efforts made to enhance biocompatibility and remodeling potential of grafts, aging related deficiencies of innate immunity result in compromised immune effector function and increased low‐level inflammation, which reduced repairing potential after damaging insult.^[^ ^27^ ^]^ Interestingly, many key roles for autophagy in macrophages become dysregulated with advanced age, which unveils a key link between autophagy and aged macrophage function.^[^ ^28^ ^]^ Tβ4 is the major actin‐sequestering molecule in all eukaryotic cells. Given that the actin networks are engaged in the early stages of autophagosome formation and membrane remodeling during autophagy,^[^ ^29^ ^]^ it is believed that Tβ4 could promote autophagy in aging immune cells. These findings, together with importance of macrophage polarization in vascular regeneration, led us to investigate whether Tβ4 might act as the immune cell rejuvenating signal and its effect on VPCs during vascular regeneration in aging body.

As a highly conserved 43‐amino acid, water‐soluble peptide, Tβ4 is rapidly metabolized and unstable in vivo, even localized and sustained release was administrated, which compromises improvement on tissue repair.^[^ ^30^ ^]^ Here we produced a recombinant human Tβ4 dimer that contains two complete Tβ4 molecules.^[^ ^31^ ^]^ We revealed an essential role for Dimeric thymosin β4 (DTβ4) in regulating all three layers of neoartery (endothelium, muscular media, adventitia) when it was incorporated into PCL nanofibrous sheath around PGS graft, and identified the adult perivascular adipose tissue (PVAT) as the dominant source of vascular progenitors which, when stimulated by DTβ4, migrate and differentiate into smooth muscle and endothelial cells. Moreover, the ability of DTβ4 to promote M2 macrophage polarization further promotes SMCs differentiation and contributes significantly to muscular remodeling. DTβ4 exhibited enhanced vascular regenerative activity when incorporated into highly tensile PCL sheath as compared with Tβ4, and effectively ameliorates aging‐induced defective regeneration, thus provide a rational strategy for clinical translation of vascular grafts.

## 2 Results

### 2.1 Perivascular Adipose Provides Vascular Forming Cells for Arterial Regeneration in Aging Rats

**Figure 1:**
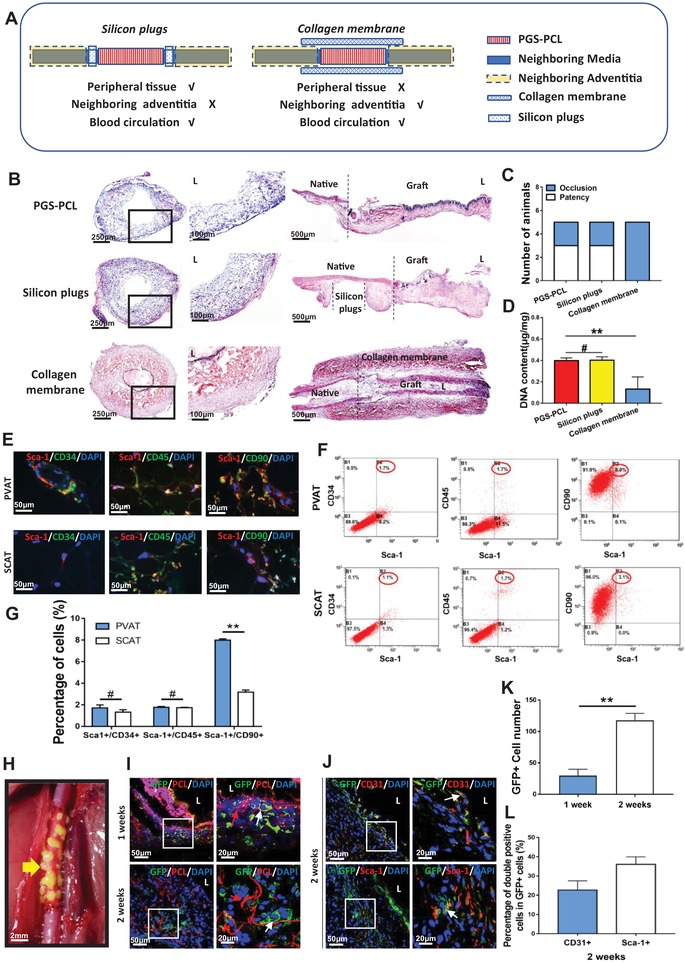
Perivascular adipose provides vascular forming cells for arterial regeneration. A) Schematic illustration of wrapping collagen membranes and placing silicon plugs. B) H&E staining of neoarteries in PGS‐PCL, silicon plus, collagen membrane groups at 2 weeks postimplantation. C) Comparison of patency rate (*n* = 5 independent samples). D) Comparison of DNA content (*n* = 3 independent samples). E) Representative immunofluorescence images of Sca‐1+/CD34+ endothelial progenitor, Sca1+/CD45+ macrophage progenitor cells, and Sca‐1+/CD90+ vascular progenitor cells in PVAT and SCAT. DAPI was used to counterstain the nuclei. F) Flow cytometry images show the percentage of Sca‐1+/CD34+ cells, Sca‐1+/CD45+ cells and Sca‐1+/CD90+ cells in PVAT and SCAT. G) Quantification of Sca‐1+/CD34+ endothelial progenitor, Sca1+/CD45+ macrophage progenitor cells and Sca‐1+/CD90+ vascular progenitor cells in PVAT and SCAT through flow cytometry analysis (*n* = 3 independent samples). H) Perivascular adipose from GFP+ rat was transplanted around PGS‐PCL graft. Yellow arrow marks the grafted PVAT from GFP+ rat. I) Representative immunofluorescence images of the cross sections of PVAT transplanted grafts displaying GFP+ cells (green) recruitment into the PCL sheath (red) at 1 week and 2 weeks postimplantation. DAPI was used to counterstain the nuclei. J) Immunofluorescence staining images show the main components of GFP+ cells identified by CD31+ (top) and Sca‐1+ (bottom) at 2 weeks postimplantation. K) The number of GFP+ cells infiltrating into the grafts at different time points (*n* = 3 independent samples). L) The proportion of CD31+/GFP+ cells and Sca‐1+/GFP+ cells in GFP+ cells, respectively (*n* = 3 independent samples). Data are represented as the mean ± SD for each group. For D) significance was determined by one‐way ANOVA followed by Tukey's post hoc analysis. For (G) and (K), significance was determined by student *t*‐test. #: *p* > 0.05, **: *p* < 0.01.

**Figure 2:**
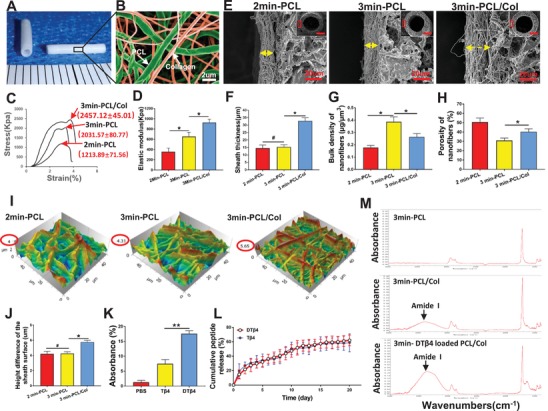
Fabrication of DTβ4 incorporated PGS‐PCL grafts. A) Photograph of DTβ4 incorporated PGS‐PCL grafts. B) SEM image shows the distribution of the two types of fibers in the hybrid PCL/Col sheath. Green indicates PCL and red indicates collagen. C) The stress–strain curves of grafts at radial directions in 2min‐PCL, 3min‐PCL, and 3min‐PCL/Col grafts (*n* = 5 independent samples). D) Comparison of the elastic modulus for above grafts (*n* = 5 independent samples). E) SEM examination of grafts shows the microstructure, which visualizes the thickness and bulk density of the sheath in the cross sections in 2min‐PCL, 3min‐PCL, and 3min‐PCL/Col grafts. Insets are the low‐magnification images. F–H) Comparison of the thickness, bulk density, and porosity of the sheath (*n* = 5 independent samples). I) 3D images show the surface roughness of sheathes of 2min‐PCL (left), 3min‐PCL (middle), and 3min‐PCL/Col (right) grafts through atomic force microscopy (AFM). J) Comparison of surface roughness among 2min‐PCL, 3min‐PCL, and 3min‐PCL/Col groups by AFM (*n* = 5 independent samples). K) Comparison of the absorbance of Micro‐FTIR spectra (3023–3677 cm^−1^) in 3min‐PCL, 3min‐PCL/Col, and 3min‐DTβ4 loaded PCL/Col groups (*n* = 3 independent samples). **2** L) Releasing profile of DTβ4 peptide from the DTβ4 loaded PCL/Col‐PGS graft (red) and Tβ4 peptide from the Tβ4 loaded PCL/Col‐PGS graft (blue) (*n* = 3 independent samples). M) Representative Micro‐FTIR spectra of 3min‐PCL, 3min‐PCL/Col, and 3min‐DTβ4 loaded PCL/Col grafts. Data are represented as the mean ± SD for each group. For (D,F–H,J,K), significance was determined by one‐way ANOVA followed by Tukey's posthoc analysis. #: *p* > 0.05, *: *p* < 0.05, **: *p* < 0.01.

Aging rats (18–20 months old) are used so that they are more representative to clinical cases. According to previous procedure,^[^ ^20^ ^]^ 2 min electrospinning enables formation of 17.6 ± 2.07 µm thickness PCL nanofibers around PGS graft. However, grafts with such sheath caused severe aneurysm (3/7) and vessel ruptures (2/7) in vivo, except 2 occluded grafts, owing to compromised remodeling potential in aging body (Figure S1a and Table S1, Supporting Information). We therefore enforced the sheath by extending PCL electrospinning time to 3 min, which produced nanofiber sheath with 18.40 ± 1.52 µm in thickness but significantly increased bulk density (0.28 ± 0.02 µg µm^−3^) (Figure 2A–H). The modified structure effectively ameliorated risk of aneurysm (1/7) at 4 weeks postimplantation (Figure S1 and Table S1, Supporting Information). To identify dominant tissue contributing rapid cell recruitment for PGS‐PCL graft in aging rats, specifically designed sheath structures are further constructed on PGS grafts (Figure S2, Supporting Information). Solid nonporous PCL sheath prevent transmural migration of abluminal cells, which significantly reduced cellularization as compared with porous PCL sheath grafts (Figure S2d and S3b,c, Supporting Information), and resulted all grafts occluding in 2 weeks after implantation (5/5) (Figure S3d, Supporting Information), in contrast to 60% patency rate in porous PGS‐PCL grafts (3/5) (**Figure** **1**C). This finding suggests majority of cells recruited are abluminally derived. Perivascular tissues include adipose around grafts and adventitia of host vessels, we thus designed shielding of collagen membranes or silicon plugs (Figure 1A,B) to separate PVAT and neighboring adventitia, respectively. Surprisingly, blocking adjacent adventitia did not reduce cell recruitment (Figure 1D) and patency rate as compared with PGS‐PCL grafts (3/5) (Figure 1C). However, collagen membrane shielded grafts acquired limited remodeling (Figure 1D), and adventitia from adjacent vessels only covered anastomosing sites (Figure 1B). Poor remodeling caused by PVAT blocking leads occluding of grafts in 2 weeks (0/5) (Figure 1C). These findings confirm that perivascular adipose, rather than adjacent adventitia is major tissue for host remodeling of grafts in abdominal aorta model. Luminal coverage of endothelial cells (ECs) presented as CD31 immunofluorescent staining revealed solid PCL sheath impeded ECs migration (Figure S4a, Supporting Information), whereas adventitia blocking with silicon plugs did not influence the grafts' endothelialization (Figure S4a,b, Supporting Information), suggesting perivascular adipose was the dominant source for graft's endothelialization.

The PVAT of abdominal aortas from aging rats (18–20 months old) were carefully dissected and immunofluorescent stained for detecting vasculogenic cells (Figure 1E). Sca‐1+/CD45+ macrophage progenitor cells and Sca‐1+/CD34+ endothelial progenitor cells were sparsely distributed in PVAT, which is comparative to subcutaneous adipose tissue (SCAT) from aging rats. Sca‐1+ cells in PVAT were more easily observed than in SCAT (Figure 1E). The immunophenotypic profiles of stromal cells were quantitatively detected by flow cytometric analysis (Figure 1F). Similar to SCAT, PVAT showed small amount of Sca‐1+/CD45+ macrophage progenitor cells and Sca‐1+/CD34+ endothelial progenitor cells (Figure 1G). Different from SCAT, most cells are positively stained for Sca‐1+/CD90+ in PVAT (Figure 1G), which is regarded as VPCs.

Transplanting perivascular adipose of abdominal aorta from green fluorescent protein transgenic rats (GFP+ rats) confirmed the cell source for vascular remodeling (Figure 1H). GFP+ cells migrated from transplanted adipose, and infiltrated into the PCL sheath since 1 week postimplantation (Figure 1I). 2 weeks' incubation enabled homing of more GFP+ cells than 1 week (Figure 1I,K). In these GFP+ cells, CD31+ cells accounted for 22.6 ± 4.88% and Sca‐1+ cells accounted for 36.0 ± 3.94%, respectively (Figure 1J,L). Coimmunostaining of SM‐MHC with GFP confirmed that differentiation of SMCs is scarce at 7 days, whereas sparsely detected at 2 weeks in grafts (Figure S15a–c, Supporting Information), which suggests GFP+ stem cells differentiate toward SMCs since the second week.

### 2.2 DTβ4 Incorporated PGS‐PCL Grafts is Highly Porous and Peptides‐Controlled Releasing

The modified, peptides loaded graft consists of a sodium citrate‐PGS mixing tube and a 25 µm thin electrospun sheath wrapping outside (**Figure** **2**A). Sodium citrate was used so that water immersion procedure including salt leaching and heparinization were avoided, thus eliminated peptides loss. A nanohybrid fibrous structure consisting of PCL nanofibers (930–1050 nm) and collagen nanofibers (550–630 nm) could be clearly identified by double labeling showing PCL fibers in green and collagen in red (Figure 2B). Notably, supplementing collagen nanofibers in sheath enhanced sheath thickness (Figure 2E,F), while make sheath looser (Figure 2E,G,H). Mechanically, adding collagen nanofibers significantly increased elastic modulus and ultimate tensile strength of the grafts (Figure 2C,D). Surface roughness examined by atomic force microscopy (AFM) reveals contacting area with perivascular tissues, which was increased with collagen incorporation (Figure 2I,J). Coelectrospinning collagen effectively carry DTβ4, which was revealed with intensified density of amide (3023–3677 cm^−1^) peaks (Figure 2K,M). The loading amount of DTβ4 or Tβ4 is about 691.8 ng cm^−1^ on the peptide loaded PCL/Col‐PGS graft according to calculation of collagen weight. As shown in Figure 2L, releasing profiles of DTβ4 and Tβ4 determined by ELISA showed that DTβ4 or Tβ4 loaded PCL/collagen sheath exhibited continuous release of the peptides over 20 d (62.07 ± 3.01% of loading amount for DTβ4 groups; 59.32 ± 4.71% of loading amount for Tβ4 groups).

To investigate the activity of peptides released from the nanofibrous sheath, we isolated PVAT explants and assessed the ability of Tβ4 and DTβ4 released from PGS‐PCL composites to induce outgrowth of stromal cells, through “Trans‐well” systems (Figure S5a,c, Supporting Information). All explants demonstrated virtually outgrowth of stromal cells (Figure S5b, Supporting Information). Significantly more cells presented in Tβ4 (130.3 ± 16.74 cells mm^−2^) and DTβ4 (265.1 ± 23.03 cells mm^−2^) groups as compared to the phosphate buffer saline (PBS) group (76.7 ± 13.15 cells mm^−2^) (Figure S5b,e, Supporting Information). The DTβ4 group increased cell migration as compared with Tβ4 and PBS group, while the migrating distance of Tβ4 group was similar to PBS group (Figure S5f, Supporting Information). Both DTβ4 and Tβ4 increase the Sca‐1+ cell migration as compared with PBS (Figure S5c,d,g, Supporting Information). In addition, DTβ4 presented stronger promoting effect on Sca‐1+ stem cell migration than Tβ4 group (Figure S5d,g, Supporting Information). Immunostaining demonstrated that the PVAT consisted mostly of VPCs (Sca‐1+) (81.8 ± 3.75%) and myofibroblasts (Vim+/MHC−) (11.2 ± 5.13%) (Figure S6a,b, Supporting Information). For ECs differentiation, cells cocultured with Tβ4 or DTβ4 incorporated PCL sheath showed tube formation (Figure S6c, Supporting Information). Significantly higher branch density and branch length are presented in DTβ4 group (Figure S6d, Supporting Information). SM‐MHC+ cells are scarce in all groups (Figure S6e,f, Supporting Information). These data indicate that DTβ4 released from scaffolds accelerate stem cell migration and endothelial cells differentiation.

DTβ4 contains 2 molecules of Tβ4, twice amount of molecular functional groups are included in its double “peptide sequence.” Administrating two peptides at same molar concentration may cause the bias of biological effects. The concentration screening study suggested endothelial differentiation of progenitors stimulated with Tβ4 was even weaker than DTβ4 at the equal molar concentration (5 vs 10 ng mL^−1^) (Figure S6c–f, Supporting Information). The same trend was also presented in “Trans‐well cell migration” test (1 vs 2 mg mL^−1^), and half molar dosage of DTβ4 (2 mg mL^−1^) exerts much promoting effect on migration of PVAT derived stem cells than Tβ4 group (2 mg mL^−1^) (Figure S5d–g, Supporting Information), which well‐illustrated synergistic effect of dimer itself. We therefore adjusted amount of DTβ4 and Tβ4 by using same mass concentration to acquire equal functional molecular groups, which may claim the superiority of dimers more accurately.^[^ ^32^ ^]^

### 2.3 Accelerated Migration of PVAT Derived ECs Upon DTβ4 Action Underlies Enhanced Patency Rates of Peptides Loaded Grafts

Longitudinally aligned ECs reduce platelet activation and adhesion, preventing intimal hyperplasia, which is essential for long‐term antithrombogenicity. Although high patency rates (80%) has been acquired in PGS‐PCL grafts,^[^ ^20^ ^]^ we surprisingly found that DTβ4 incorporated grafts presented 100% patency rates (10/10) (Figure S7b, Supporting Information) even in aging rats. This correlates with almost confluent endothelium (75.2 ± 4.08%) visualized by CD31 immunostaining just 2 weeks after DTβ4 grafts implantation (Figure S7a,c, Supporting Information). In contrast, intermittently ECs monolayer covering of the lumen was presented in Tβ4 or PBS grafts (Figure S7a,c, Supporting Information). Full endothelialization in DTβ4 grafts (92.6 ± 4.15%) was acquired after 4 weeks, which is much faster than partial endothelialization in Tβ4 grafts (73.2 ± 3.49%) and PBS grafts (62.4 ± 4.72%) (Figure S7a,c, Supporting Information). Detailed luminal morphology was revealed by scanning electron microscope (SEM) examination. Apart from the dogma that ECs may migrate from the anastomosed vessels, ECs clusters scattering on luminal surface indicates other cell sources (Figure S7d,e, Supporting Information). Obviously, DTβ4 incorporated sheath enabled faster ECs coverage in all segments of grafts both at 2 and 4 weeks after implantation, while mild and small thrombi are sporadically detected on bare PCL fibrous sheath of PBS or Tβ4 grafts (Figure S7d,e, Supporting Information).

The intermediate filament protein “Nestin” is expressed during embryonic development, but considered largely restricted to areas of angiogenesis or neurogenesis in the adult.^[^ ^33^ ^]^ It has been shown to be constitutively, and highly‐selectively expressed in neonatal ECs^2^. At 2 weeks after implantation, we identified sporadical distribution of Nestin+ cells within the neoadventitia of all grafts (Figure S7f, Supporting Information), and quantitatively, DTβ4 grafts (105.2 ± 11.12/view) recruited more Nestin+ cells as compared with Tβ4 (56.4 ± 5.89/view) and PBS (51.0 ± 7.17/view) grafts (Figure S7h, Supporting Information). At 4 weeks, inward migrating Nestin+ cells were localized within the medial and luminal layer of all grafts and significantly more cells formed circumferential band in DTβ4 grafts (212.6 ± 17.33/view) than that in Tβ4 (53.6 ± 9.39/view) and PBS grafts (54.2 ± 6.72/view) (Figure S7g,h, Supporting Information). Immunostaining at 4 weeks suggested that Nestin+ cells were capable of turning into endothelial cells, which was revealed by colocalization of Nestin with CD31 in these three groups (Figure S7g,h, Supporting Information). Quantitatively, more Nestin+/CD31+ cells presented in DTβ4 group (105 ± 13.28/view) than that in Tβ4 (18.4 ± 6.46/view) and PBS groups (18.8 ± 5.49/view) (Figure S7i, Supporting Information). These results indicate that DTβ4 accelerated grafts endothelialization through mobilizing more endothelial progenitor cells from PVAT and promoted their inward migration. Furthermore, Immunostaining of Sca1+ (green)/CD31(red) in cross‐sections of DTβ4 loaded grafts at 2 weeks (Figure S16, Supporting Information) suggests that part of PVAT‐derived Sca‐1+ cells (green) are costained with CD31(red). Together with in vitro findings that the endothelial differentiation of PVAT‐derived Sca‐1+ cells after stimulus by DTβ4 (Figure S6c,d, Supporting Information), it is confirmed at least part of regenerated endothelium is from PVAT‐derived Sca‐1+ cells.

### 2.4 DTβ4 Promoted Muscular Remodeling in Endogenous Arterial Regeneration

**Figure 3:**
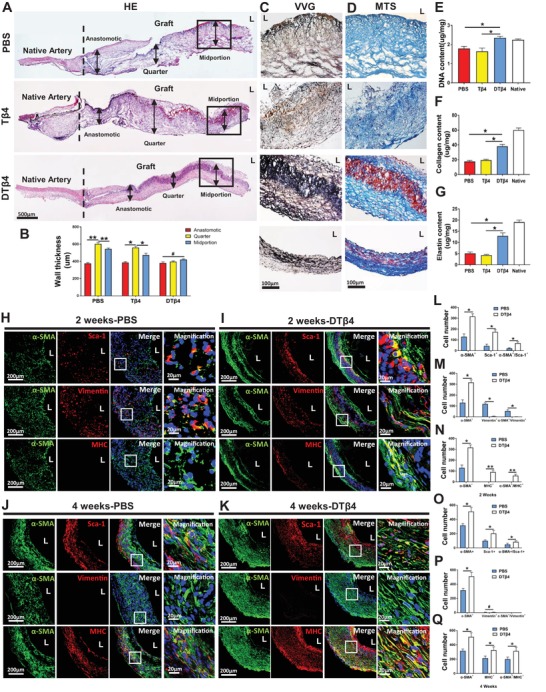
DTβ4 Promoted muscular remodeling in endogenous arterial regeneration. A) H&E staining of longitudinal section of neoarteries in PBS, Tβ4, and DTβ4 groups at 4 weeks postimplantation. B) Average layer thickness of anastomotic, quarter, and midportion from neoarteries in PBS, Tβ4, and DTβ4 groups at 4 weeks postimplantation (*n* = 3 independent samples). C) Magnified views of Verhoeff‐Van Gieson (VVG) staining reveal elastic fibers (black) alignment in the midportion of neoarteries in PBS, Tβ4, and DTβ4 loaded grafts at 4 weeks postimplantation. D) Magnified views of Masson's trichrome (MTS) staining show collagen (blue) content in the midportion of neoarteries from PBS, Tβ4, and DTβ4 loaded grafts at 4 weeks postimplantation. E) Comparison of DNA content (*n* = 3 independent samples). F,G) Quantification of elastin and collagen respectively (*n* = 3 independent samples). H–K) Immunofluorescence staining of cross‐sections of neoarteries reveals the recruitment of α‐SMA+(green)/MHC+(red) SMCs, α‐SMA+(green)/vimentin+(red) myofibroblasts, and the costaining of Sca‐1+ cells(red) and α‐SMA+ cells(green) in PBS and DTβ4 loaded grafts at 2 and 4 weeks postimplantation. DAPI was included to counterstain the nuclei. L,O) Quantification of α‐SMA+, Sca‐1+, and α‐SMA+/Sca‐1+ cells among PBS and DTβ4 loaded grafts at 2 and 4 weeks postimplantation (*n* = 3 independent samples). M,P) Quantification of α‐SMA+, Vimentin+, and α‐SMA+/Vimentin+ cells among PBS and DTβ4 loaded grafts at 2 and 4 weeks postimplantation (*n* = 3 independent samples). N,Q) Quantification of α‐SMA+, MHC+, and α‐SMA+/MHC+ cells among PBS and DTβ4 loaded grafts at 2 and 4 weeks postimplantation (*n* = 3 independent samples). Data are represented as the mean ± SD for each group. For B,E–G), significance was determined by one‐way ANOVA followed by Tukey's post hoc analysis. For (I–Q), significance was determined by student *t*‐test. #: *p* > 0.05, *: *p* < 0.05, **: *p* < 0.01.

Despite limited influence of DTβ4 on myogenic differentiation of PVAT derived progenitor cells in vitro (Figure S6e,f, Supporting Information), in vivo results confirmed DTβ4 enhanced muscular remodeling including elastin and contractile SMCs in neoarteries at 4 weeks after implantation (**Figure** **3**A,C,D). Remodeling vessel wall in such graft presented compactly aligned, trilayer structure and even thickness, whereas remodeling vessel walls of Tβ4 and PBS grafts presented obscure, loose structure as well as variant thickness (Figure 3A,D). DNA quantification revealed the highest cell density in DTβ4 grafts (2.35 ± 0.07 µg mg^−1^) as compared with PBS (1.79 ± 0.11 µg mg^−1^) or Tβ4 (1.64 ± 0.17 µg mg^−1^) incorporated grafts (Figure 3E). Consistent with the hematoxylin and eosin (H&E) staining, Masson trichrome staining (MTS) revealed wave‐shape collagen fibers in DTβ4 graft wall, and regularly aligned with similar thickness (Figure 3D). VVG staining revealed a large amount of elastic fibers circumferentially formed around PCL sheath in DTβ4 group, whereas few elastic fibers formed around PBS or Tβ4 incorporated sheath (Figure 3C). Quantitative data showed that both elastin and collagen produced in DTβ4 group were significantly higher than those in Tβ4 or PBS group at 4 weeks (Figure 3F,G), which was believed to contribute stronger strength for neoarteries.

Homing sufficient SMCs in vascular grafts is essential for muscular arterial regeneration. Although many recruited cells expressing a‐SMA+ markers, they may be myofibroblasts (α‐SMA+/ Vimentin+). Thus, we further analyzed the expression of vimentin and myosin heavy chain (MHC). MHC is a late‐stage differentiation marker for smooth muscle, which indicate a contractile smooth muscle phenotype. In PBS loaded PCL/Col‐PGS grafts (PBS), immunofluorescent staining revealed the most a‐SMA+ cells also costained Vimentin at 14 d, indicating myofibroblasts (Figure 3H,M). In addition, small number of Sca‐1+ progenitor cells were found sparsely distributed in grafts (Figure 3H). Some Sca‐1+ cells are also coimmunostained with a‐SMA, indicating transition into SMCs (Figure 3H). With progressively host remodeling, more SMCs identified as costaining of a‐SMA and SM‐MHC emerged in grafts, whereas myofibroblasts diminished at 28 d (Figure 3J). Sca‐1+ cells also increased at 28 d, and distributed transmurally (Figure 3H). Surprisingly, when DTβ4 is incorporated into the grafts, the spatiotemporal distribution of myofibroblasts and SMCs changed significantly (Figure 3I,K). Although amount of myofibroblasts in DTβ4 loaded grafts remains the same level with PBS loaded grafts at 14 d (Figure 3H,I,M), more SMCs and Sca‐1+ cells infiltrated (Figure 3H,I,L,N) and increasingly occupied DTβ4 loaded grafts at 28 d (Figure 3J,Q). Spatially, Sca1+ cells preferentially concentrated around DTβ4 incorporated nanofibers, bright‐field image revealed that a band of dark fibers mixed with cells in this region, which is different from scattering distribution in control groups (Figure S8, Supporting Information).

### 2.5 DTβ4 Efficiently Leads to the M2 Polarization of PVAT Residential Macrophages

**Figure 4:**
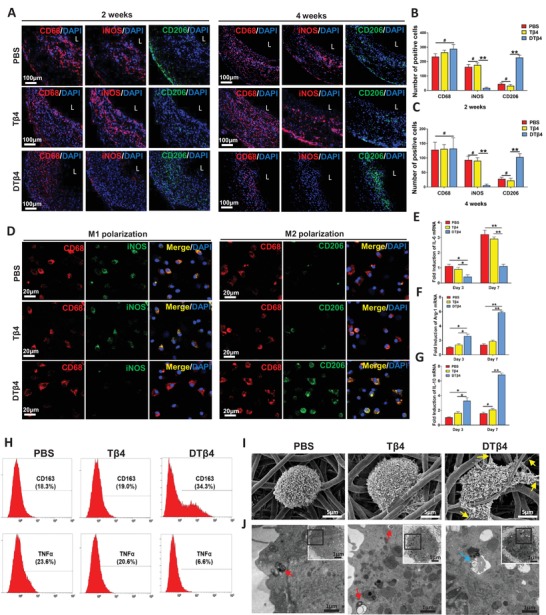
DTβ4 efficiently leads to the M2 polarization of PVAT residential macrophages. A) Representative immunfluorescence of macrophage polarization on cross‐sections of neoarteries in PBS, Tβ4, and DTβ4 groups. At each time point, enhanced number of CD68+/CD206+ M2 macrophages were observed in the DTβ4 group, while CD68+/iNOS+ M1 macrophages were dominant in the PBS and Tβ4 group. L: lumen. B,C) Number of CD68, iNOS, and CD163 positive cells quantifies total macrophage number, M1 type and M2 type macrophages number, respectively, among PBS, Tβ4, and DTβ4 groups at 2 and 4 weeks postimplantation (*n* = 3 independent samples). D) Immunofluorescent staining of macrophages with CD68 (red), CD206 or iNOS (green), and nuclei (blue) after 7 d culturing on PBS, Tβ4, or DTβ4 incorporated scaffolds. The DTβ4 graft was occupied with more CD68+/CD206+ M2 macrophages, whereas more CD68+/iNOS+ M1 macrophages were detected in the PBS and Tβ4 grafts. L: lumen. E–G) Relative mRNA expressions of macrophage polarization‐related genes at 3 and 7 d. M2 macrophage polarization‐related anti‐inflammatory genes (IL‐10, Arg‐1) were significantly upregulated in the DTβ4 group at each time point. However, the M1 macrophage‐related proinflammatory gene (IL‐6) expression level was much lower in the DTβ4 group (*n* = 3 independent samples). H) Flow cytometry reveals enhanced expression of M2 macrophage marker CD163 and a decreased expression of M1 macrophage marker TNF‐α in the DTβ4 group on day 7 as compared with PBS and Tβ4 grafts. I,J) Morphology and nanostructure examination of macrophages seeded on PBS, Tβ4, and DTβ4 incorporated scaffolds by SEM and TEM. Yellow arrows: pseudopods, red arrows: heterolysosomes, blue arrows: autophagolysosomes. Data are represented as the mean ± SD for each group. For (B,C,E–G), significance was determined by one‐way ANOVA followed by Tukey's post hoc analysis. #: *p* > 0.05, *: *p* < 0.05, **: *p* < 0.01.

Inflammatory pathways have been reported to induce endogenous reprograming of somatic cells.^[^ ^34^ ^]^ We suspect polarization of macrophages is necessary to myogenic differentiation of PVAT derived progenitors. Immunocytochemical studies revealed macrophages actively participate in grafts remodeling, however, distinctly spatiotemporal distribution of heterogeneous subpopulations is presented in different peptides loaded grafts (**Figure** **4**A). At 2 weeks, the number of iNOS+ macrophages (M1) and CD206+ macrophages (M2) in PBS grafts was similar to Tβ4 grafts, which showed predominant proportion of iNOS+ macrophages thus indicate proinflammatory responses (Figure 4A,B). These cells resided, although significantly decreased, until the 4 weeks postoperatively (Figure 4A,C). In contrast, the DTβ4 sheath grafts elicited typical M2 transition through 2–4 weeks, which correlates with constructive remodeling in grafts (Figure 4A–C).

To reveal the interaction between peptides and macrophages, we seeded macrophages, derived from rat bone marrow blood, on PBS, Tβ4, or DTβ4 incorporated scaffolds and analyzed the transition. M2 markers CD206, CD163, Arg‐1, and IL‐10 were examined, and iNOS, IL‐6, and TNF‐α were used for M1 macrophage identification. As immunofluorescent staining shown, after 7 days' culture, CD68+/CD163+ M2 macrophages were observed on DTβ4 incorporated scaffolds, whereas most macrophages on PBS or Tβ4 expressed M1 markers CD68 and iNOS (Figure 4D). Real time‐polymerase chain reaction (RT‐PCR) revealed that macrophages in DTβ4 group expressed much higher levels of the M2‐macrophage related genes (Arg‐1 and IL‐10) and lower levels of M1 related gene (IL‐6) at days 3 and 7, as compared with the PBS or Tβ4 group (Figure 4E,G). Flow cytometry further confirm the phenotypes of polarized macrophages, which presented 34.3% of total cells expressing M2 marker CD163 in the DTβ4 group, while 18.3% in PBS group and 19.0% in Tβ4 group. Conversely, only 6.6% of cells express M1 marker TNF‐α in DTβ4 group, whereas 20.6% in Tβ4 group and 23.6% in PBS group (Figure 4H). To further reveal the interaction between peptides incorporated nanointerface and macrophages, morphology, and ultrastructure of macrophages were examined by using SEM and transmission electron microscopy (TEM) (Figure 4I,J). Macrophages in the DTβ4 group stretched out more pseudopods (Figure 4I), while few pseudopodia were presented in macrophages adhered on PBS or Tβ4 sheaths. TEM revealed that autophagolysosomes, representative as autophagy, were more frequently observed in macrophages attached on DTβ4 loaded grafts (Figure 4J). Western blotting confirmed that microtubule‐associated protein light chain 3 β (LC3B) dependent autophagy was enhanced after DTβ4 action (Figure S9, Supporting Information). In contrast, several heterolysosomes appeared in macrophages seeded on the PBS and Tβ4 groups, which indicated the phagocytosis of macrophages against degrading scaffolds (Figure 4J). Considering the roles of macrophage autophagy played in anti‐inflammation, these results suggested that DTβ4 effectively activate macrophages and polarize them toward M2 subset mainly through stimulating autophagy.

### 2.6 Depleting PVAT Derived Macrophages Impaired Muscular Remodeling in DTβ4 Loaded Nanofibrous Interface

**Figure 5:**
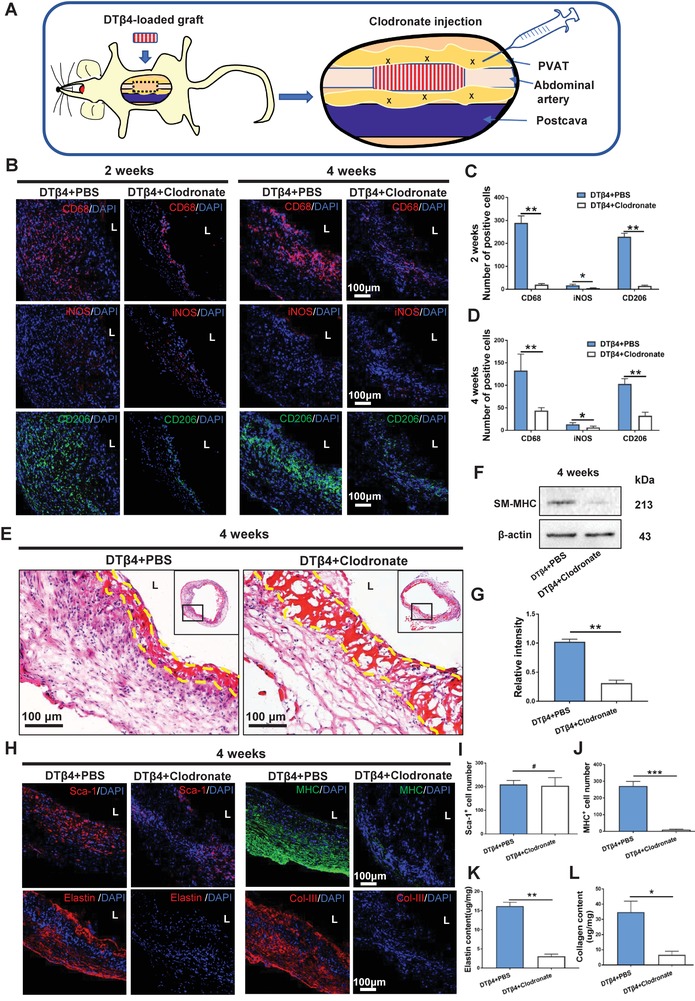
Impaired muscular remodeling in DTβ4 loaded nanofibrous interface by depleting perivascular adipose residential macrophages. A) Schematic illustration of clodronate injection. B) Representative immunofluorescent images reveals macrophage polarization in neoarteries of DTβ4+PBS and DTβ4+Clodronate groups at 2 and 4 weeks postimplantation. C,D) Quantification of CD68+, iNOS+, and CD206+ cells among DTβ4 + PBS group and DTβ4 + Clodronate group at 2 and 4 weeks postimplantation respectively (*n* = 3 independent samples). E) H&E staining of the neoarteries in DTβ4+PBS and DTβ4+Clodronate groups at 4 weeks postimplantation. Yellow dotted line marks the region of residual scaffold. F,G) Western blotting reveals SM‐MHC expression of the neoarteries in DTβ4+PBS and DTβ4+Clodronate groups at 4 weeks postimplantation (*n* = 3 independent samples). H) Representative immunofluorescent staining of Sca1+, MHC+, Elastin, and Col‐III of neoarteries. I,J) Quantification of Sca‐1+ cells and MHC+ cells among DTβ4 + PBS group and DTβ4 + Clodronate group at 4 weeks postimplantation (*n* = 3 independent samples). K,L) Quantification of elastin and collagen among DTβ4 + PBS group and DTβ4 + Clodronate group at 4 weeks postimplantation respectively (*n* = 3 independent samples). Data are represented as the mean ± SD for each group. For (C,D,G,I–L), significance was determined by student *t*‐test. #: *p* > 0.05, *: *p* < 0.05, **: *p* < 0.01.

In vivo clodronate liposome treatment depleted macrophages in PVAT efficiently (**Figure** **5**A). CD68+ macrophages in the DTβ4+clodronate group was dramatically reduced as compared with the DTβ4+PBS group at 2 weeks, confirming the validity of monocyte/macrophage depletion (Figure 5B,C). M2 macrophage marker CD206 was significantly downregulated in the DTβ4 + Clodronate group compared with DTβ4 + PBS group at 2 and 4 weeks (Figure 5B–D). Same trend was detected in the iNOS+ M1 macrophages in DTβ4 + Clodronate group and DTβ4 + PBS group at both time points (Figure 5B–D). Interestingly, SM‐MHC+ contractile cells were greatly diminished in grafts upon clodronate treatment (Figure 5E,H,J). Western blot confirmed that SM‐MHC expression of neoarteries in the DTβ4 + clodronate group was significantly reduced compared with that in the DTβ4 + PBS group at 4 weeks (Figure 5F,G). Interestingly, the amount of Sca‐1+ cells were similar between two groups (Figure 5H,I), suggesting that macrophage did not influence recruitment PVAT derived stem cells.

Functional extracellular matrix (ECM) deposition is essential for arterial physiological function. After 4 weeks' remodeling, ECM deposition in the neoarteries was obviously different upon macrophages depleting. Immunostaining presented substantial amount of elastin in DTβ4+PBS group (16.15 ± 1.03 µg mg^−1^), which aligned circumferentially and mimicked their orientation in native artery, while very small amount of elastin presented in the DTβ4+Clodronate group (3.08 ± 0.54 µg mg^−1^) (Figure 5H,K). Immunofluorescence further revealed that production of collagen III in remodeling DTβ4+PBS grafts was stronger than that in remodeling DTβ4+Clodronate grafts (Figure 5H,L). Taken together, both in vivo and in vitro findings demonstrate that M2 macrophage polarization induced by DTβ4 interacts with Sca‐1+ stem cells to promote myogenic differentiation and elastin formation.

It should be also noted that all grafts in DTβ4+Clodronate group are patent when harvested at 4 weeks (*n* = 3). DTβ4 grafts combined with perivascular clodronate injection presented comparable endothelium coverage with DTβ4 loaded grafts (Figure S13a–c, Supporting Information), which suggests DTβ4 accelerated endothelialization is less related with macrophages. Together with in vitro and in vivo results, DTβ4 induced endothelial differentiation of PVAT derived stem cells, and promoted myogenic differentiation of progenitors through polarizing macrophages.

### 2.7 M2‐Polarized Macrophages Promoted Myogenic Differentiation of Sca‐1+ Stem Cells In Vitro

**Figure 6:**
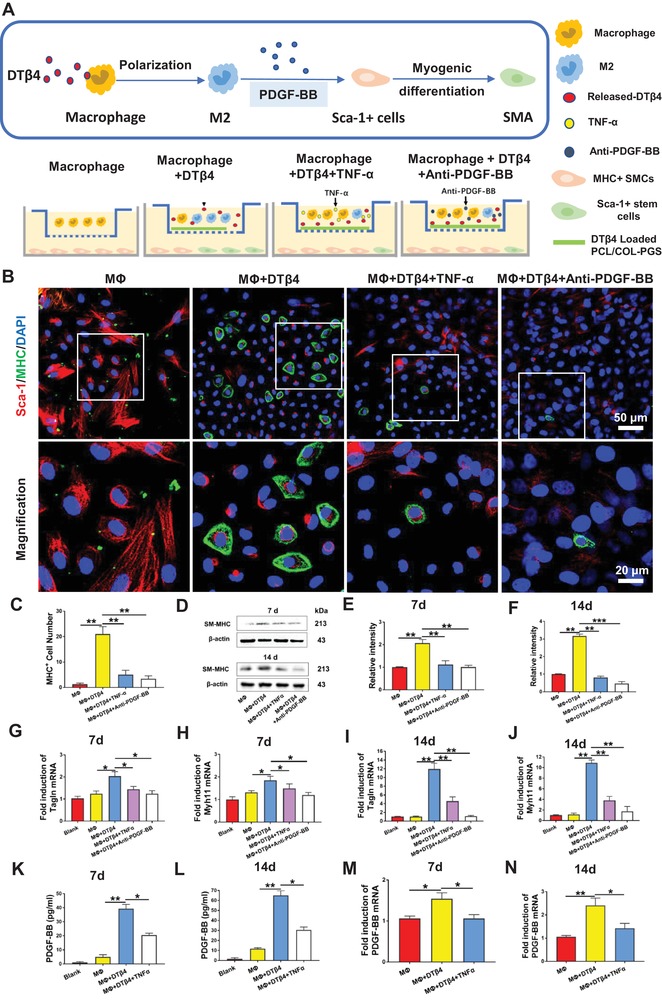
M2‐Polarized Macrophages promoted myogenic Differentiation Sca‐1+ stem cells by secreting PDGF‐BB. A) Schematic illustration of Sca‐1+ stem cells cocultured with macrophages seeded on nanofibrous scaffolds. B) Immunofluorescent staining of Sca‐1+(red) stem cells, MHC+(green) SMCs, and nuclei (blue) after 7 d coculturing with macrophages in MΦ, MΦ+DTβ4, MΦ+DTβ4+TNF‐α, and MΦ+DTβ4+Anti‐PDGF‐BB groups. C) Comparison of the number of MHC+ cells in MΦ, MΦ+DTβ4, MΦ+DTβ4+TNF‐α, and MΦ+DTβ4+Anti‐PDGF‐BB groups (*n* = 5 independent samples). D–F) Western blotting reveals SM‐MHC expression in Sca‐1+ stem cells on days 7 and 14 (*n* = 3 independent samples). G–J) Relative mRNA expressions of Tagln and myh11 in Sca‐1+ cells cocultured with macrophage stimulated by different condition for 7 and 14 d (*n* = 3 independent samples). Blank: Sca‐1+ cells cultured with unconditioned medium. K,L) Quantification of PDGF‐BB secreted by macrophages in MΦ, MΦ+DTβ4, and MΦ+DTβ4+TNF‐α groups at 7 and 14 d (*n* = 3 independent samples). M,N) Relative mRNA expressions of PDGF‐BB in macrophages after different treatments for 7 and 14 d (*n* = 3 independent samples). Blank: macrophages cultured with unconditioned medium. mRNA expression of Tagln, myh11, and PDGF‐BB were analyzed by RT‐qPCR using the ∆∆CT method. Data are represented as the mean ± SD for each group. For (C,E–N), significance was determined by one‐way ANOVA followed by Tukey's post hoc analysis. *: *p* < 0.05, **: *p* < 0.01.

To further investigate how DTβ4 incorporated nanointerface influenced macrophage polarization and direct Sca‐1+ stem cell differentiation, we analyzed myogenic differentiation of Sca‐1+ stem cells cocultured with macrophages seeded on nanofibrous scaffolds through transwell system (**Figure** **6**A). Sca‐1+ cells cocultured with the scaffolds and macrophages exhibited a highly branched, elongated morphology (Figure 6B). Myogenic differentiation of stem cells was revealed by increasing number of SM‐MHC+ cells. Quantitatively, the number of SM‐MHC+ cell in MΦ+DTβ4 group was significantly higher than that in MΦ group at 14 d (Figure 6C). This trend was further confirmed by the protein levels of SM‐MHC as well as mRNA expression of Tagln and myh11 at 7 and 14 d (Figure 6D–J).

To explore the mechanism that M2 macrophages drive Sca‐1+ cells to differentiate into SMCs, we assessed the concentrations of a variety of cytokines, including platelet‐derived growth factor‐BB (PDGF‐BB), transforming growth factor‐β1 (TGF‐β1), basic fibroblast growth factor, and vascular endothelial growth factor (VEGF) in supernatant from the macrophages seeded DTβ4 incorporated scaffolds (Figure 6K–N; and Figure S10, Supporting Information). Notably, compared with supernatant from the MΦ group, the concentrations of PDGF‐BB were significantly elevated at 7 and 14 d in the supernatant from the MΦ+DTβ4 group (Figure 6K,L). This trend was confirmed by mRNA expression of PDGF‐BB at both time points (Figure 6M,N). In contrast, the concentration of the other three cytokines were not altered in the supernatant from these two groups (Figure S10, Supporting Information). Conversely, adding PDGF‐BB antibody in supernatant correlated with lack of SMC differentiation, which is proved by immunostaining, western blotting for SM‐MHC, and RT‐PCR for mRNA expression of Tagln and myh11 (Figure 6B–J). Additionally, adding TNF‐α, activator for M1 transition of macrophages, significantly reduced the concentration and mRNA expression of PDGF‐BB at both 7 and 14 d (Figure 6K–N). Furthermore, mRNA expression of Tagln and myh11 and the protein levels of SM‐MHC at 7 and 14 d demonstrated that adding TNF‐α obviously decreased myogenic differentiation of Sca‐1 cells (Figure 6D–J). These findings specifically revealed that DTβ4 incorporated nanofibrous interface could facilitate macrophage polarization to M2 subset, which in turn promotes myogenic differentiation of PVAT derived stem cells through secreting PDGF‐BB.

### 2.8 DTβ4 Incorporated PGS‐PCL Grafts Ameliorates Aging‐Induced Impaired Muscular Remodeling

**Figure 7:**
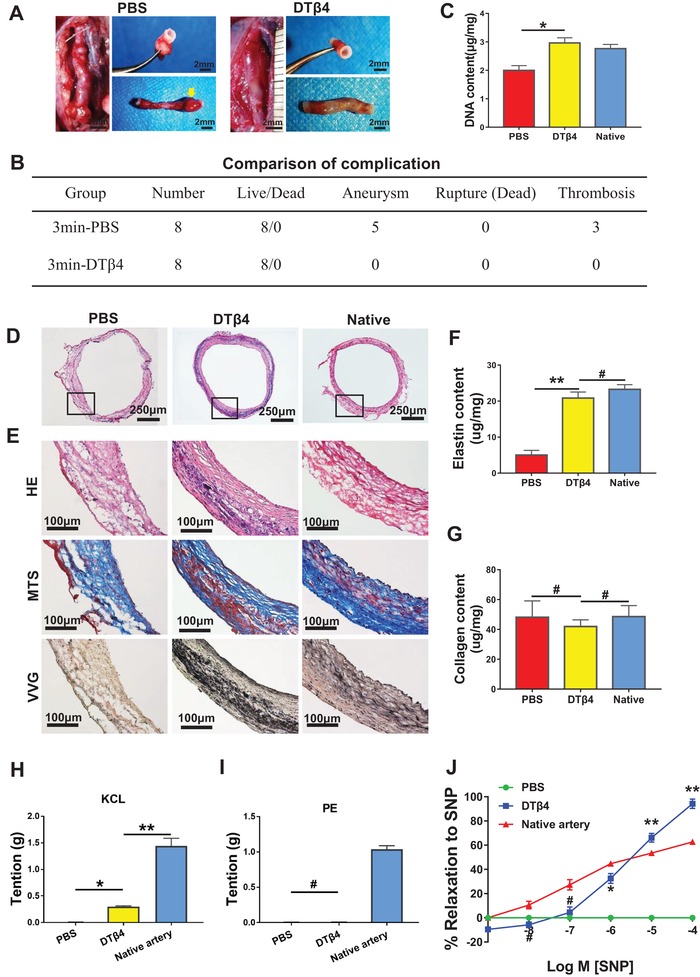
DTβ4 incorporated PGS‐PCL grafts ameliorates aging‐induced impaired regenerative remodeling. A) Transition from grafts into neoarteries in 3min‐PBS incorporated PGS‐PCL grafts and 3min‐DTβ4 incorporated PGS‐PCL grafts through 3 months. Yellow arrow marked the aneurysm. B) Comparison of complication among 3min‐PBS and 3min‐DTβ4 groups (*n* = 8 independent samples). C) Comparison of DNA content (*n* = 5 independent samples). D) H&E staining of neoarteries from 3min‐PBS and 3min‐DTβ4 groups and native aorta. E) Magnified views of H&E, VVG, and MTS staining of neoarteries in these two groups and native aorta. F) Quantification of elastin (*n* = 5 independent samples). G) Quantification of collagen (*n* = 3 independent samples). H) Neoarteries demonstrated constriction in response to potassium chloride (KCL). The magnitude of constriction of neoarteries in 3min‐DTβ4 group was less than native arteries, but significantly higher than that in 3min‐PBS group. I) Neoarteries demonstrated very weak constriction in response to the selective α1‐adrenergic receptor agonist phenylephrine (PE). J) Neoarteries respond to the vascular smooth muscle cell specific activator sodium nitroprusside (SNP) in a dose‐dependent manner. Data are represented as the mean ± SEM for each group. Results of (H,I,J) are the mean ± SD of *n* = 12 rings from 3 animals per treatment group. For (C,F–I), significance was determined by one‐way ANOVA followed by Tukey's post hoc analysis. For (J), comparison between PBS and DTβ4 groups was determined by student *t*‐test. #: *p* > 0.05, *: *p* < 0.05, **: *p* < 0.01.

Aging greatly impairs remodeling potential of vessel wall,^[^ ^35^ ^]^ mainly owing to quantitative difference in stem cells between adult and neonatal tissues. We investigated whether locally released DTβ4 would improve PGS‐PCL grafts based arterial regeneration in aging animals. Accordingly, incorporating DTβ4 successfully eliminate the occurrence of aneurysm in aging rats at 3 months postimplant (**Figure** **7**A,B). Although increasing sheath density reduced cell recruitment into nanofibrous sheath, this trend was significantly reversed by incorporating DTβ4 (Figure 7C). Histological analysis underlies the distinct muscular remodeling in these groups. Increasing PCL sheath density improved mechanical stability, however, neointima is uneven and predominantly presented fibrotic components, such as COL‐I (Figure 7D,E). In contrast, DTβ4 loaded grafts regenerated even and continuous muscular intima, which was characterized with circumferentially aligned collagen and elastic fibers (Figure 7D,E). Immunofluorescent staining confirmed that I, III collagen and elastin in DTβ4 group were spatially, coordinately located in trilayer structures (Figure S11a, Supporting Information). Marked difference of histology was further revealed in ECM quantification. At 3 months, the elastin content was 5.02 ± 1.29 µg mg^−1^ in PBS group, significantly lower than that of DTβ4 group (Figure 7F). This finding indicated DTβ4 rejuvenates endogenous elastin production from SMCs in aging individuals. The collagen production of these groups is at the same level, which is also close to native aorta (Figure 7G).

We used myography to determine whether regenerated SMCs respond vasomotor signals. After 3 months, neoarteries from DTβ4 group displayed apparent vascular function of contraction and relaxation, indicating that vascular cells within neoarteries express functional phenotypes (Figure S11b, Supporting Information). The magnitude of neoarteries from DTβ4 group constricted in response to potassium chloride (KCL) was much higher than that in PBS group, though it was less than that of native arteries (Figure 7H). Neoarteries from both DTβ4 and PBS group were little responsive to phenylephrine (Figure 7I). Neoarteries from DTβ4 group also dilated in response to endothelium‐independent vasodilators SNP, while PBS group failed to response (Figure 7J). These data suggested that DTβ4 accelerated the recovery of regenerated vascular cells in expressing healthy phenotypes.

## 3 Discussion

One of the essential prerequisites for arterial regeneration is the identification of endogenous vascular progenitors and their niche that can be targeted by new therapeutic approaches. Adjacent vessel derived endothelium, circulating smooth muscle or bone marrow progenitor cells are regarded to contribute remodeling of vascular grafts.^[^ ^36^, ^37^ ^]^ Additionally, adventitia derived VPCs, representative as Sca‐1+ cells are isolated and proven to enabled neointima formation in injured vessels or synthetic grafts.^[^ ^38^ ^]^ Different from slowly degrading polymers in above studies, PGS‐PCL grafts recruited significant amount of progenitor and inflammatory cells in 14 d postimplantation.^[^ ^7^ ^]^ Tracing their origin may reveal how to accelerate their recruitment. In this study, our design departs from previous studies in that we separately examined roles played by adjacent adventitia and PVAT. GFP positive adipose transplantation model visualized interaction between degrading scaffolds and infiltrating PVAT cells. Cells recruited are concentrated in PCL nanofibers, whereas diminished with degrading of PGS, thus confirmed pivotal role of nanofibers‐PVAT cells interaction in neoartery. To the best of our knowledge, affirming the decisive contribution of PVAT derived cells, rather than adventitia, in fast degrading polymer mediated arterial regeneration is new to vascular substitutes. This report is the first step that identifying the dominant tissue offering functional cells for biodegrading polymer guided arterial regeneration.

Ultrathin and nanofibrous sheath provides opening channels for cell migration, which enables fast cell recruitment. However, apart from hydrogel or porous sponges, ultrathin nanofiber sheath does not favor the peptides loading, mainly owing to much exposure to body liquid and rapid metabolism of small molecules. Here, although in vitro proangiogenic potential of Tβ4 has been detected as previously reported,^[^ ^23^, ^30^ ^]^ we failed to detect its promoting role in accelerating endotheliarization in vivo, which confirmed the peptides loading deficiency in nanofibrous interface. In contrast, DTβ4 loaded grafts acquired 100% patency rates in rat model, which is significantly higher than all other grafts. Profound alternation in recruiting cell subpopulations as well as endothelialization suggests durable bioactivity of dimers in nanofiber interface. Although further mechanism remains elusive, we believe extended half‐life and slower metabolism rates of DTβ4 owing to larger molecular weight play roles. Apart from traditional opinion that reendothelization may rely on ECs migration from anastomosed vessels, we ascribed fast, full luminal endothelialization to short distance between PVAT derived ECs and luminal surface of PCL sheath. It is also known that proliferating macrophages secrete angiogenic factors that stimulate neovascularization. Some studies revealed acquisition of high angiogenesis‐inducing capacity by human and murine macrophages requires their polarization toward the M2 phenotype, which accelerates the migration of ECs through production of matrix metalloproteinase‐9.^[^ ^39^ ^]^ Departing from these findings, in macrophage depleting model, it is confirmed that DTβ4 accelerated endothelialization is less related with macrophages. Owing to the profound sources of ECs, ECs differentiated from PVAT derived Sca‐1+ cells may only account for part of the regenerated endothelium. Reendothelialization of vascular grafts and detailed mechanism remain to be further investigated. It is known that vascular growth is regulated by interactions between the endothelium and the surrounding cells.^[^ ^40^ ^]^ However, we failed to found the contribution of accelerated endothelialization on SMCs recruitment in PGS‐PCL graft. CD31 immunostaining for clodronate treated DTβ4 grafts at 4 weeks has shown that accelerated endothelialization fail to amend compromised SMCs recruitment caused by macrophages depletion (Figure S13, Supporting Information). We further compared SMCs recruitment between PCL grafts and PGS‐PCL grafts in young rats published before.^[^ ^19^ ^]^ Both grafts share the same microenvironment and acquire full endothelization at 4 weeks, while the SMCs recruitment in PCL graft is very limited as compared with PGS grafts (Figure S14, Supporting Information). Taken together, we believe the contribution of regenerated endothelium to SMCs recruitment is limited in biodegrading grafts.

Accumulating evidence suggests biomaterials may influence the recruitment of progenitor cells through regulating immunoreaction. Macrophages from local tissues, especially M2 subset is proven to play pivotal role in bone regeneration, skin repair, and myocardium regeneration.^[^ ^41^, ^42^, ^43^ ^]^ Embryonic‐derived resident tissue populations facilitate the resolution of inflammation and instruct tissue repair in some organs.^[^ ^44^ ^]^ Although circulation and adventitia are known as the source of macrophages, tracing cell source by using GFP+ tissues confirmed PVAT resident macrophages, play pivotal role in regenerating muscular artery. This outside‐interior remodeling style emphasized central role of PCL nanofibrous interface, in mediating profound PVAT‐biomaterial interaction that leads to myogenic differentiation. Although physical structures, such as fiber diameter or fiber density influences M2 transition of macrophages,^[^ ^45^ ^]^ both in vitro and in vivo results in this study revealed DTβ4 incorporation is determinant to M1/M2 ratio conversion. For the first time, we found that DTβ4 treated macrophages presented more autophagosomes, indicating enhanced autophagy of cells. We speculated that the anti‐inflammatory effect of DTβ4 is achieved via autophagy‐mediated control of macrophage polarization. The crucial role of autophagy in regulating macrophage polarization has been widely studied in cancer. Recent studies highlight the role of selective autophagy in regulating the nuclear factor‐κB (NF‐κB) pathway, which plays a central role in the regulation of macrophage polarization in tumor microenvironment.^[^ ^46^ ^]^ The enhanced macrophage autophagy enables anti‐inflammation to promote tissue repair in response to cutaneous injury.^[^ ^47^ ^]^ This process might closely relate to the expressions of autophagy proteins which inhibits proinflammatory cytokine production.^[^ ^48^ ^]^ Besides, it remains elusive why dimer owns stronger biological activity than monomer both in vitro and in vivo. One possible explanation is that cellular receptors on macrophages are prone to bind dimers rather than monomer. Further identification of the receptor of Tβ4 on macrophages would be helpful to reveal the mechanism. In summary, our results indicate DTβ4 could modulate the transition of CD206+ (M2) macrophages, and could be of tremendous clinical value.

Although Tβ4 directly‐induced VSMCs differentiation of mural cells during embryogenesis,^[^ ^49^ ^]^ In vitro coculture here revealed both Tβ4 and DTβ4 stimulated proliferation of myofibroblast rather than SMCs differentiation in adult PVAT derived cells. We believed stemness and differentiating potential of progenitor cells may vary greatly from embryo to adult. Interestingly, we found M2 macrophages‐induced VSMCs differentiation of Sca‐1+ cells, and DTβ4 loaded nanofiber sheath accelerated M2 polarization early at 14 D postimplantation. It is known that chemokines released from M2 macrophages, such as PDGF and IGF,^[^ ^42^, ^50^ ^]^ dramatically enhanced homing efficiency and differentiation of progenitor cells, in vitro study here revealed M2 macrophage derived PDGF‐BB plays critical role in myogenic differentiation of PVAT derived stem cells. Large amount of Sca‐1+ cells and Nestin+ cells concentrated in nanofibrous sheath since 14 D, which enabled sufficient muscular remodeling of grafts. After early inflammatory phase subsides, the predominant macrophage population assumes a wound healing phenotype that is characterized by the production of numerous growth factors including PDGF, TGF‐β1, IGF‐1, and VEGF‐α, that promote cellular proliferation and blood vessel development.^[^ ^44^, ^51^, ^52^ ^]^ Although multiple signals likely influence myogenic differentiation of Sca‐1+ progenitor cells, our study highlights the importance of Sca‐1+ cells/macrophage interactions and, particularly, of DTβ4 in promoting recruitment of vascular progenitor cells through fine‐tuning the number of CD206+ macrophages. These data correspond with the function of macrophages in wound healing. Recruiting more macrophages is vital to subsequent myogenic progenitors.

Impaired host remodeling is always accompanied with aging. It has been shown that VPCs in PVAT are significantly reduced in aged body, which may delay tissue repair.^[^ ^35^ ^]^ Our study presented that medium PCL density leads to the aneurysm and vessel burst in tested rats, thus claimed the necessity of applying high PCL density sheath in grafts in aging body. As expected, DTβ4 loading significantly enhanced muscular remodeling of grafts, without perioperative complications, which assured clinical applicability in aging individuals. When these composite DTβ4‐Collagen‐PCL nanofibers were implanted into defects, the myofibroblasts and ECs from surrounding PVAT would be activated and mobilized to the spaces among nanofibers upon the biological activity of the DTβ4, which may amend impaired mobilizing potential of these cells in aging body. Porous PGS tube provides biodegrading stent for nanofibrous sheath, however, conventional salt leaching as well as heparinization process may sacrifice part of loaded peptides. We thus use sodium citrate as raw material for salt template, and formed antithrombogenic PGS stent. For further large animal studies, fabricating large size PGS‐sodium citrate composite may resort to 3D printing techniques, which may lead to advanced biomaterials and graft designs that can bring synthetic small arterial grafts closer to clinical translation.

## 4 Conclusion

**Figure 8:**
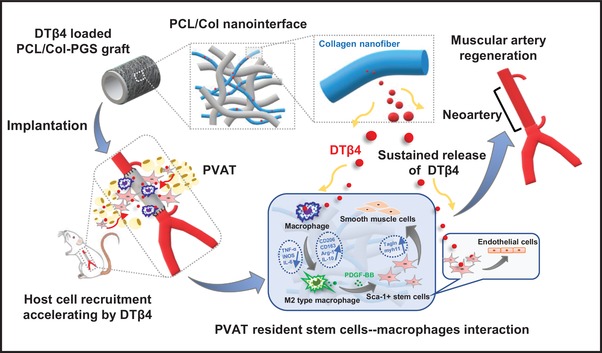
Schematic illustration of how perivascular adipose stem cells–macrophage interaction modulated by Dimeric thymosin β4 loaded nanofibrous interface enhanced regeneration of muscular artery.

We identified the adult PVAT as the dominant source of vascular progenitors in rat abdominal aorta model, and clarified the mechanism by which DTβ4 enhances arterial muscle remodeling in aging body. Different from conventional synthetic grafts, DTβ4 incorporated grafts exhibits enhanced vascular regenerative activity and effectively ameliorates aging‐induced defective regeneration, thus provide a rational strategy for clinical translation of vascular grafts (**Figure** **8**).

## 5 Experimental Section

Detailed methods are provided in the Supporting Information.

## Conflict of Interest

The authors declare no conflict of interest.

## Supporting information

Supporting Information
