# Supplementary material for: Dimeric Thymosin β4 Loaded Nanofibrous Interface Enhanced Regeneration of Muscular Artery in Aging Body through Modulating Perivascular Adipose Stem Cell–Macrophage Interaction
Source: Adv Sci (Weinh). 2020 Mar 16;7(8):1903307. doi: 10.1002/advs.201903307 (PMC7175290; doi:10.1002/advs.201903307)
Supplement: Supplementary file 1 — Supporting Information [file ADVS-7-1903307-s001.pdf]

Copyright WILEY-VCH Verlag GmbH & Co. KGaA, 69469 Weinheim, Germany, 2020.

ADVANCED SCIENCE
Open Access

Supporting Information

for *Adv. Sci.,* DOI: 10.1002/advs.201903307

Dimeric Thymosin β4 Loaded Nanofibrous Interface Enhanced Regeneration of Muscular Artery in Aging Body through Modulating Perivascular Adipose Stem Cell–Macrophage Interaction

Wanli Chen, Sansan Jia, Xinchi Zhang, Siqian Zhang, Huan Liu, Xin Yang, Cun Zhang, and Wei Wu**

Supporting Information

Dimeric thymosin β4 loaded nanofibrous interface enhanced regeneration of muscular artery in aging body through modulating perivascular adipose stem cells--macrophage interaction

Wanli Chen[1†], Sansan Jia[1†], Xinchi Zhang[1], Siqian Zhang[1], Huan Liu[3], Xin Yang[1], Cun Zhang[2*], Wei Wu[1*]

Materials and Method

Fabrication of grafts with different sheath microstructure and biological activity. To fabricate anti-thrombogenic PGS tube, sodium citrate instead of NaCl (Figure S12, Supporting Information), was fabricated into salt template as previously described [1]. Co-electrospinning was employed to prepare the hybrid PCL/collagen (PCL/Col) sheath on the PGS-salt core using two separate spinnerets according to following steps. First, PCL (Mn 80 kDa; Aldrich, MO, USA) was dissolved in 2,2,2-trifluoroethanol at 14% weight/volume (w/v) and collagen (Collagen type I from bovine achilles tendon, Sigma) was dissolved in hexafluoroisopropanol (HFIP) 8% w/v, respectively. The peptide DTβ4 was synthesized and purified as previously described [2]. In brief, two entire complementary DNA sequences of Tβ4 optimized with E. coli-preferred codons were synthesized by Bio Asia Diagnostics, Inc., (Shanghai,China), and were constructed into prokaryotic expression plasmid pET22b(+) (Promega Corporation, Fitchburg, WI, USA) by NdeI and SalI restriction enzymes. Then the pET22b-DTβ4 plasmid was transformed into E. coli BL21 (DE3; Promega) using the calcium chloride method. A 10% (weight/volume) bacteria suspension acquired by Large-scale cultivation was used for the purification. After a simple hydrophobic interaction chromatography (HIC) purification, the purity of DTβ4 reached 98%. Tβ4 (4963.49 Da; ChinaPeptides Ltd., Shanghai) and DTβ4 (9,961.0 Da) peptides were then dissolved in deionized water at room temperature to produce 2.0 mg/mL solution of peptides, respectively. After that, collagen and peptide solutions were mixed at 4:1 (v/v). PBS was used instead of Tβ4 and DTβ4 solution in the control group. The PCL solution (2.5ml/h) and collagen solution(1ml/h) with or without peptides were co-electrospun onto a rotating PGS-salt template at 120 RPM for 3 min. As the results, three kinds of grafts with varying bulk density were acquired. Composite grafts were lyophilized, sterilized with ultraviolet irradiation for 60 mins and stored at -80℃ until use.

Characterization of scaffold. The scaffolds were characterized for structure, mechanical properties, distributions of the chemical components, and releasing patterns of loaded peptides. The cross-sections and outer surface of sheath were examined by scanning electronic microscopy (Hitachi, s-4800, Japan), and sheath thickness was measured. Using the equations as previously described [20], porosity and bulk density of the PCL and PCL/Col sheaths of the grafts were calculated. For measurement of tensile strength and elastic modulus, the scaffolds were cut into 2 mm long segments, and were fixed with two same hooks connected to the loading cell. Uniaxial tensile force was applied to each segment at a rate of 2 mm/min with initial force of 0.05 N until rupture.

In addition, the nanostructure and nanomechanics of scaffolds were investigated by AFM (Dimension Icon, Bruker, USA) using a relative comparison method at room temperature. The samples were assembled onto freshly cleaved mica, and three scans were performed in each sample. The obtained property maps with 512 × 512 pixels were analyzed using a NanoScope 14.12 software. For every map, five regions were randomly chosen, and the median value was calculated as representative value.

To test the chemical components and spatial mineral/matrix distribution in different scaffolds,

3min-PCL, 3 min-PCL/Col and DTβ4 loaded 3min PCL/Col sheath were deposited on optical metal base, and then scanned at a resolution of 4 cm^{-1} using a FTIR Nicolet iS50 spectrometer (Thermo, USA). Collection of the spectra was performed using OMNIC 7.3 (Thermo Electron Corporation).

Finally, we tested the releasing patterns of PCL/Col sheath. Briefly, DTβ4 or Tβ4 loaded scaffolds (1 cm) were incubated in RPMI-1640 (1 mL; containing 1% BSA) at 37°C for 20 days with shaking at 50 rpm. The incubated medium was collected and changed every day. The amount of peptide released from each group was measured using ELISA kit (Cat#EKU07666, BIOMATIK). The amount of DTβ4, loaded on the 3min PGS-PCL/Col scaffold (1cm), was calculated with the following equations:

$$g_{Col} = g_{3min\text{-}PCL/Col} - g_{3min\text{-}PCL}$$

$$g_{DT\beta4} = (V_{DT\beta4} \times C_{DT\beta4})/(V_{Col} \times C_{Col}) \times g_{Col}$$

The $g_{3min\text{-}PCL/Col}$ is the weight of 3min-PCL/Col graft. The $g_{3min\text{-}PCL}$ is the weight of 3min-PCL graft. Both of them were measured by an electronic scale. The g_{Col} is the amount of spraying collagen on 3min-PCL/Col graft. $V_{DT\beta4}$ means the volume of the DTβ4 solution, and V_{Col} means the volume of the collagen solution. $V_{DT\beta4}$: V_{Col} =1:4 as previously mentioned. $C_{DT\beta4}$ means the concentration of the DTβ4 solution and $C_{DT\beta4}$=2 mg/ml as previously mentioned. C_{Col} means the concentration of the collagen solution and C_{Col}=80 mg/ml as previously mentioned. The amount of Tβ4, loaded on the 3min PGS-PCL/Col scaffold (1cm), was calculated in the same way.

Animal grouping and surgery. GFP transgenic aging rat (18-20-month-old, 550-600g) were purchased from Cyagen Biosciences Inc. (GuangDong, China). Sprague Dawley aging rats (18-20-month-old, 550-600g) were purchased from Fourth military medical university laboratory animal center, Xi'an. All animal procedures were approved by the Animal Experiments Ethical Committee of Fourth Military Medical University and complied with the Guide for Care and Use of Laboratory Animals.

Interpositional implantation of the 2min-PGS-PCL (n=7) and 3min-PGS-PCL (n=7) grafts were conducted in aging rats' abdominal aortas as previously described [20]. Four weeks after implantation, animals were sacrificed to explant neoarteries for ex-vivo analysis. Then we designed a method to study the source of fast recruited cells by setting up different blocking groups. In brief, total 25 aging (18-20-month-old) rats were separated into five groups: 1) Collagen membrane (n=5): after 3min-PGS-PCL grafts anastomoses with the artery, the collagen membranes were wrap around the grafts to block the perivascular adipose tissue. 2) Silicon plus (n=5): to block the adventitia of adjacent vessels, the silicon plugs were wrapped around the autologous arteries after 3min-PGS-PCL grafts were anastomosed with the artery. 3) PGS nonporous-PCL(n=5): through microwaving the 3min-PGS-PCL graft for 5 minutes with medium baking temperature, a sealed sheath was formed to prevent the transmural cell migration. 4) Bilayer PCL (n=5): the bilayer PCL graft had a tough PCL core fabricated by casting a PCL prepolymer solution into a fused salt template. 5) The 3min-PGS-PCL (n=5) was set as control. Two weeks after implantation, animals of each group were anesthetized to explant neoarteries for ex-vivo analysis, and then sacrificed by injecting overdosage of pentobarbital sodium (Sigma, USA).

The PVAT of GFP aging rats was transplanted to SD aging rats to investigate whether PVAT was the main source of fast recruited cells (n=10). Briefly, a dissection microscope was used to collect 10mg of PVAT from the inferior margin of the lesser curvature of the thoracic aorta of donor GFP rats. Then, the collected PVAT, cut into little pieces on ice, was transplanted on the surface of post-implanted grafts. At predetermined time points (1st and 2nd weeks after transplantation), animals were anesthetized to explant neoarteries for ex-vivo analysis.

To evaluate the effect of peptides loaded grafts, a total of 46 aging (18-20-month-old) SD rats (500-550g) were assigned randomly into three groups and implanted with 3min-PBS (n=18), 3min-Tβ4 (n=10) and 3min-DTβ4 (n=18) loaded grafts respectively. Animals were sacrificed at the planned time points (the 2nd, 4th and 12th weeks after implantation), then the neoarteries were explanted for further experiments.

Clodronate liposomes (50mg/kg, Netherlands) was used to deplete the monocytes/macrophages in PVAT. The perivascular adipose tissue of aging rats in experimental group (n=6) was injected with clodronate liposomes at multiple points after DTβ4-loaded scaffold was implanted. The aging rats in control group (n=6) received equal liposome-encapsulated PBS injection at the same locations. All of the rats without additional injection were sacrificed at 2 weeks and 4 weeks post-implantation, then the neoarteries were explanted for further experiments.

Histological analysis of explanted vascular grafts. In order to perform histochemical staining, the grafts embedded in OCT (Sakura Finetek, CA, USA) were rapidly frozen with liquid nitrogen, and then cross sectioned into 6 μm thick pieces. After fixed with 4% paraformaldehyde for 5 min, the sections were stained with hematoxylin and eosin (H&E), Masson's trichrome and Verhoeffe-Van Gieson (VVG), and then observed under upright microscope (Nikon55i, Japan). For SEM analysis, the prepared samples were mount onto aluminium stubs and then sputter-coated with gold. Subsequently, SEM (Hitachi, s-4800, Japan) was used to analyzed the samples.

Biochemical evaluation. To measure the elastin content of regenerated and native arteries, the total elastin of each explants was extracted and measured using a Fastin Elastin assay (F2000; Biocolor, Carrickfergus, UK) according to manufacturer's instructions, then pooled and normalized to sample wet weight to yield the mass of insoluble elastin per tissue wet weight (μg/mg). For collagen quantification, total collagen of each explants was quantified by Sircol™ Collagen Assay kit. Collagen concentration in pooled supernatants was measured following the kit instruction and insoluble collagen per wet weight of each sample was calculated from the collagen standard curve. DNA quantification: We thawed and minced frozen explants, and then incubated for 6 h in lysis buffer (Qiagen) and proteinase K (12 mAU reaction-1) at 56℃. DNA was isolated from samples using manufacturer's instructions. Then the DNA content was measured by absorbance at 260 nm and normalized to tissue wet weight.

Immunofluorescence staining. Immunofluorescence staining was performed as previously described. To identify the PVAT derived stromal cells, the macrophage progenitor cells were co-stained using rabbit anti-Sca-1 (Millipore, Germany) and mouse anti-CD45 (GeneTex, USA), the mesenchymal stem cells were co-stained using rabbit anti-Sca-1 and mouse anti-CD90

(Abcam, UK), and the endothelial progenitor cells were co-stained using rabbit anti-Sca-1 and mouse anti-CD34 (Abcam, UK).

To investigate transplanted GFP+ cells infiltrating the sheath, the PCL solution was mixed with Alexa Fluor 594 goat anti-rabbit at 4:1 (v/v) before electrospun. Endothelial cell staining was performed using rabbit anti-CD31 (Novus, USA) primary antibody. The smooth muscle cells were stained using mouse anti-α-SMA (GeneTex, USA) and mouse anti-SM-MHC (Abcam, UK) primary antibodies. The endothelial precursor cell was performed using mouse anti-Nestin (Abcam, UK). The fibroblasts were stained using rabbit anti-Vimentin (GeneTex, USA). For elastin and collagen staining, slides were incubated with rabbit anti-Elastin (Abcam, UK), mouse anti-Collagen I (Abcam, UK) and mouse anti-Collagen III (Abcam, UK). To observe inflammatory cells in the explanted grafts, mouse anti-CD68 (Abcam, UK), rabbit anti-iNOS (Abcam, UK) and rabbit anti-CD206 (Abcam, UK) were used as primary antibodies. After incubated overnight at 4 ℃, slides were then washed two times with PBS solution and incubated with the respective fluorescein isothiocyanate-conjugated or tetramethylrhodamine isothiocyanate-conjugated secondary antibody for 60 min at 37 °C, followed by 5 min of nuclear staining with DAPI. The samples were observed with Olympus Fluoview 1000 confocal microscope (Japan). Tissue slides pretreated without primary antibody, were used as negative, and rat aortas were used as positive controls. Different cell populations were determined based on cell counts from each immunostained images on 6 different parts (respectively: 12, 2, 4, 6, 8, 10 o'clock position). The data were collected from three different rats in each group. Details of primary antibodies are listed in Table S2 (Supporting Information).

Cell Culture. Monocytes derived from rat bone marrow blood were induced to differentiate into macrophages by macrophage colony stimulating factor (M-CSF) for 7 days at 37 °C with 5% CO_2. The 24-well plates of 15 mm diameter with PBS, Tβ4, DTβ4 loaded PCL/Col-PGS films were sterilized under ultraviolet light for 2 h before use. After monocytes derived macrophage adhered on these three groups, cells were cultured for 3 and 7 days for detection of macrophage polarization.

To investigate whether macrophages in response to DTβ4 loaded scaffolds could regulate muscular differentiation of PVAT-derived Sca-1+ cells, rat PVAT-derived Sca-1+ cells seeded in 6-well plates were cocultured with rat bone marrow derived macrophages seeded on DTβ4 loaded PCL/Col-PGS films in the upper chamber (pore size, 0.4 μm; Thermo Scientific). As control, macrophages were seeded on PCL-PGS films in upper chamber. Besides, TNF-α (10ng/ml, PeproTech) and PDGF-BB antibody (20ng/ml, Bio-techne) were added to the culture medium at the beginning of coculturing respectively. After cocultured for 7 days and 14 days, Sca-1+ cells under different cocultured condition were collected respectively for the further analysis.

Flow Cytometry. Flow cytometry was performed to identify the PVAT derived stromal cells. Breifly, the adipose tissues, cut into small pieces, were digested with 0.2% I-collagenase (Sigma, Aldrich) at 37°C for 45 min, and then the cell suspension was filtered through nylon cell strainer (Falcon) twice to remove tissue debris. The stromal cells were further resuspended in red blood cell lysis buffer (eBioscience) for 10 min and incubated in 1% bovine serum albumin (BSA) containing fluorochrome conjugated antibodies directed or indirected against the following cell

surface markers: CD45, Sca-1, CD90, and CD34. In addition, the surface markers of M1/M2 macrophages were examined by flow cytometry. The macrophages cells were isolated by trypsinization after cultured on PBS, Tβ4, DTβ4 loaded PCL/Gel-PGS films for 7 days, incubated with FITC-conjugated anti-CD163 (BioLegend, USA), and PE-conjugated anti-TNF-α (GeneTex, USA) for 30 min. Finally, the FCS files exported bya BD flow cytometer (Verse) were analyzed using FlowJo 8.3.3 software (Tree Star Inc).

Quantitative Real-Time Polymerase Chain Reaction. Total RNA from cell lysates was extracted with Trizol reagent (Invitrogen), and cDNA was synthesized using Super Script III (Invitrogen) and stored at −20 °C. Real-time PCR was carried out using gene-specific primers and SYBR Green (Invitrogen) on 7900HT Fast Time PCR. The results were analyzed using the comparative threshold cycle method and normalized to endogenous reference gene Gapdh, and reported results as relative values (△△CT) to the mean gene expression of control native aorta. Primer sequences are listed in Table S3 (Supporting Information).

Western blot. Total proteins in cell lysates were harvested with a lysis solution, separated by 10% SDS-PAGE gels, and then transferred to polyvinylidene difluoride membranes and blocked in 5% nonfat milk. Subsequently, SM-MHC antibody (GeneTex, USA) and LC3B antibody (GeneTex, USA) were dropped onto the membranes and incubated overnight at 4 °C, respectively. The membranes were then treated with a horseradish peroxidase-conjugated secondary antibody, and protein bands were detected by enhanced chemiluminescence. Each experiment was performed three times to achieve comparable results. The relative density was measured using Image J 1.37v software (Wayne Rasband).

Enzyme-Linked Immunosorbent Assay. The supernatants of the co-cultured macrophages were collected at day 7 and stored at −80 °C before use. The secretion levels of PDGF-BB, TGF-β1, bFGF and VEGF were examined with ELISA kits (R&D systems) following the manufacturer's guidance.

Concentration screening assay. Rat PVAT-derived Sca-1+ cells were isolated by outgrowth of the explant method and seeded into a 24-well plate. DTβ4 was prepared at 10 ng/ml and Tβ4 was prepared at two concentrations: 5 ng/ml and 10 ng/ml, PBS was used as the control. After 24 hours peptides intervention, cells in plate were immunofluorescence stained and observed with Olympus Fluoview 1000 confocal microscope (Japan).

To investigate the effects of peptides on cell migration of PVAT, the adherent perivascular adipose tissue and cultured rat PVAT-derived Sca-1+ cells were induced with peptides incorporated scaffold in vitro. PVAT dissected from aging rats (18-20 months old) was cut into 1mm x 1mm pieces and seeded onto a 12-well plate. After 4 hours, the adherent PVAT was cocultured with PBS, Tβ4 (1mg/ml), Tβ4 (2mg/ml) and DTβ4 (2mg/ml) incorporated scaffolds respectively. After 72 h, tissue blocks were gently washed and observed under light microscope (IX71, Olympus, Japan).

Similarly, rat PVAT-derived Sca-1+ cells were isolated by outgrowth of the explant method and

cultured in α-MEM containing 10% FBS. After starved for 12 hours, 100 μl of serum-free medium containing $1x10^5$ cells were added to each of the upper chamber. Meanwhile, PBS, Tβ4 (1mg/ml), Tβ4 (2mg/ml) and DTβ4 (2mg/ml) incorporated scaffolds were incubated in the lower chamber of transwell inserts with 8.0 μm pore size membrane filters (Corning Life Science, USA). After 12 hours' incubation, non-migrated cells in the upper wells were removed from the filter by washing with PBS three times and gentle scraping with cotton swabs. Cells migrating through the pores were fixed with methanol for 15 minutes and stained with 0.1% crystal violet (Sigma-Aldrich). A light microscope (Nikon55i, Japan) was used to count the cell number on the underside of the insert. Five fields were counted for each well to estimate the mean number of cells for each replicate.

Functional arterial myography assays. After rats humanely euthanized, neoartery and native aortic artery were rapidly removed and placed in cold Krebs buffer solution consisting of (in mM) 118.3 NaCl, 14.7 KCl, 1.2 KH_2PO4, 1.2 $MgSO_47H_2O$, 2.5 $CaCl_22H_2O$, 25 $NaHCO_3$, 11.1 dextrose, and 0.026 EDTA, pH 7.40. These vessels segments (2 mm in length) were mounted in a dual wire myograph system (Danish MyoTechnology). After equilibrated for 60 min, these individual vessels were loaded to tension. Neoartery may not be as strong as native artery, so the tension started with 0.2, 0.4, 0.8 to 1.0 g (the optimized passive tension). The presence of functional smooth muscle cells (SMCs) was indicated by the contractile responses induced by adding KCI (60 mM). The function of the neo-endothelium was confirmed by the relaxation by acetylcholine (Ach; 10^{-9}–10^{-4} M) in preconstricted segment by phenylephrine (PE; 10^{-4} M), and the vascular smooth muscle function was evaluated by vascular relaxation in response to sodium nitroprusside (SNP; 10^{-9}–10^{-4} M). Nonlinear regression curves were plotted as percentage of relaxation using all points on the concentration response curve.

Statistical Analysis. Statistical analyses were performed using SPSS software, version 18.0 (IBM, Chicago, IL, http://www.ibm.com). The continuous variables were expressed as mean ± standard deviation (SD). For comparison between two groups, the means were compared using Student t-test. The difference between multiple groups was determined by one-way analysis of variance (ANOVA) followed by Tukey's post hoc analysis. $p < 0.05$ was considered statistically significant.

References

[1] K. W. Lee, Y. Stolz Db Fau - Wang, Y. Wang. *PNAS* **2011**, 108, 2705.

[2] T. J. Xu, X.-W. Wang Q Fau - Ma, Z. Ma Xw Fau - Zhang, W. Zhang Z Fau - Zhang, X.-C. Zhang W Fau - Xue, C. Xue Xc Fau - Zhang, Q. Zhang C Fau - Hao, W.-N. Hao Q Fau - Li, Y.-Q. Li Wn Fau - Zhang, M. Zhang Yq Fau - Li, M. Li. *Drug Des., Dev. Ther.* **2013**, 7, 1075.

Table S1.
Counting of complications in grafts with different sheath density.

Group	Number	Live/Dead	Aneurysm	Rupture (Dead)	Thrombosis
2min-PGS-PCL	7	5/2	3	2	2
3min-PGS-PCL	7	7/0	1	0	3

Table S2. Primary Antibodies

Primary Epitope	Dilution	Supplier	Catalog no.
Sca-1	1:500	Millipore	AB4336
CD45	1:50	Abcam	ab10558
CD90	1:500	Abcam	ab225
CD34	1:50	Abcam	ab81289
CD31	1:100	Novus	NB100-2284
α-SMA	1:50	GeneTex	GTX18147
SM-MHC	1:200	Abcam	ab212657
Nestin	1:200	Abcam	ab6142
Vimentin	1:200	GeneTex	GTX100619
Collagen I	1:2000	GeneTex	GTX26308
Collagen III	1:1000	Abcam	ab6310
CD68	1:200	Abcam	ab201340
iNOS	1:100	Abcam	ab15323
CD206	1:200	Abcam	ab64693
Elastin	1:200	Abcam	ab21610
LC3B	1:1500	GeneTex	GTX127375
SM-MHC	1:1000	GeneTex	GTX39681
CD45	1:80	BioLegend	202207
CD90	1:800	BioLegend	206105
Sca-1	1:50	Biorbyt	orb9766
TNF-α	1:80	BioLegend	506104
CD163	1:10	GeneTex	GTX43731

Table S3. qPCR Primers

Gene	Forward	Reverse
VEGF	TGAACTTTCTGCTCTCTTGGG	GATGTCCACCAGGGTCTCAAT
TGF-β	AAGGACCTGGGTTGGAAGTGG	CGGGTTGTGTTGGTTGTAGAG
bFGF	ATCAAGGGAGTGTGTGCGAAC	TTCGTTTCAGTGCCACATACC
Arg-1	GGACATCGTGTACATCGGCT	CTTCCTTCCCAGCAGGTAGC
IL-10	CCTCTGGATACAGCTGCGAC	GTAGATGCCGGGTGGTTCAA
IL-6	AGTTGCCTTCTTGGGACTGATGTTG	GGTATCCTCTGTGAAGTCTCCTCTCC
Tagln	GGAAACCCACCCTCTCAGTC	TGCACTAGCCAAGTCATCCG
Myh11	TGCACTAGCCAAGTCATCCG	AGGCAGTTGATGGTTGGGAG
Gapdh	AGTGCCAGCCTCGTCTCATA	GACTGTGCCGTTGAACTTGC

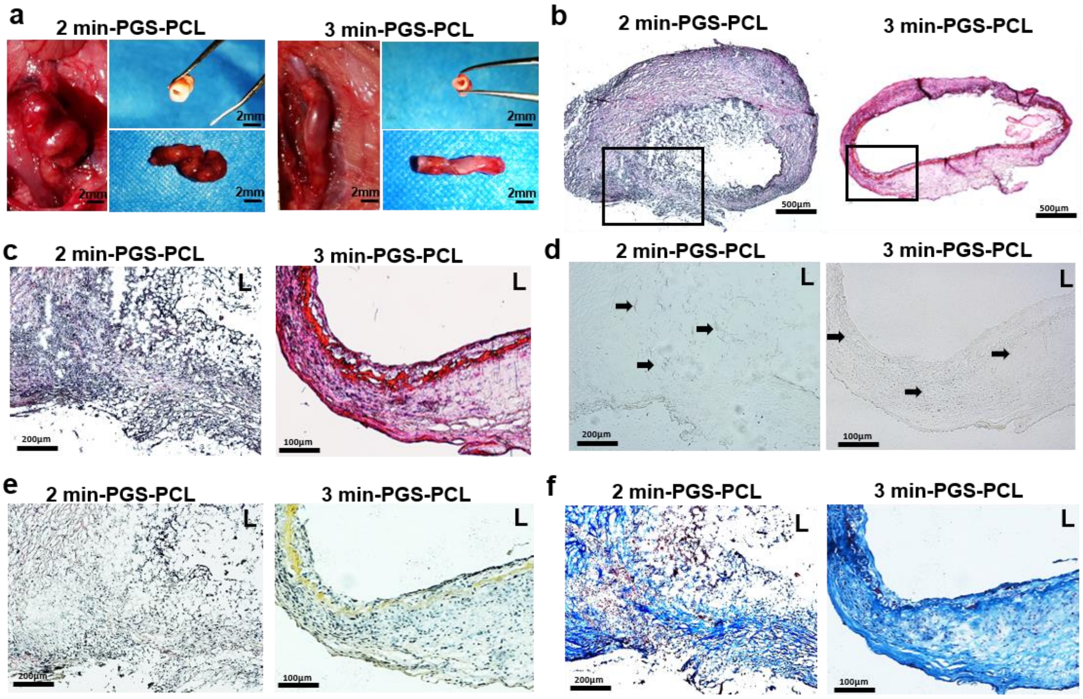

Figure S1. Tissue remodeling of PCL nano-fiber sheath with different density in aging rats. a, Transition from grafts into neoarteries in 2min-PGS-PCL and 3min-PGS-PCL groups through 4 weeks. **b**, H&E staining of neoarteries from these groups. **c**, Magnified views of H&E staining of neoarteries in these groups. **d**, Bright-field images show the distribution of residual nanofibers in the neoarteries of 2min-PGS-PCL and 3min-PGS-PCL groups at 4 weeks post-implantation respectively. Black arrows mark the residual nanofibers. **e and f**, Magnified views of VVG and MTS staining of neoarteries in 2min-PGS-PCL and 3min-PGS-PCL groups at 4 weeks post-implantation. L: lumen.

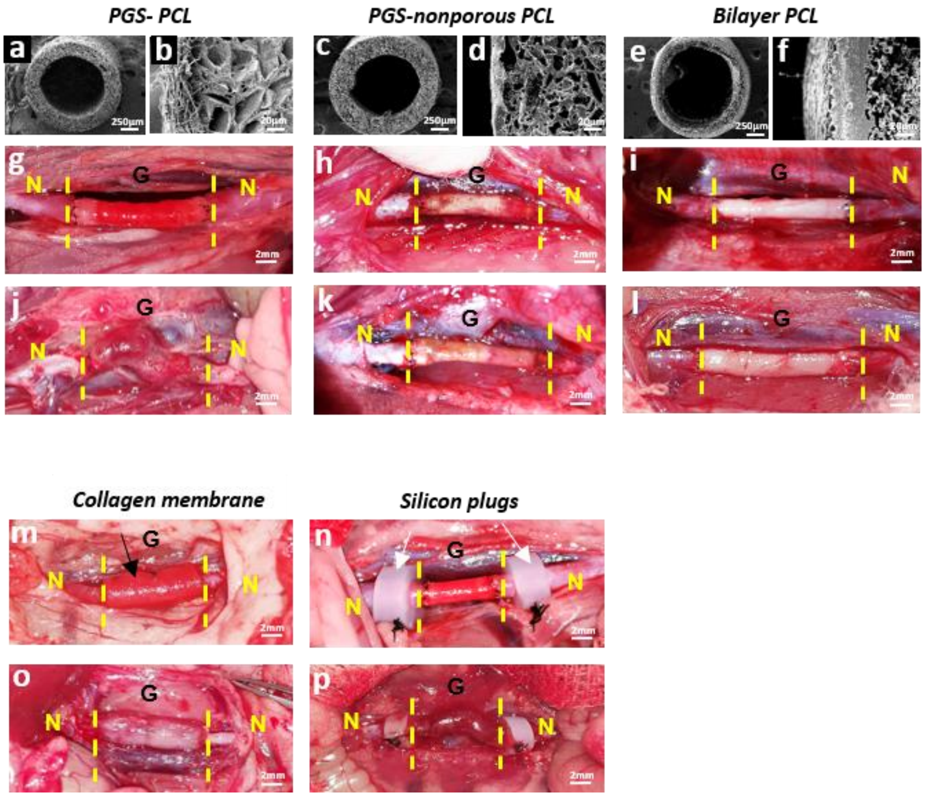

Figure S2. PGS-PCL grafts with different physical shields and sheath structures. a, **c and e**, SEM images present the top view of the whole 3min-PGS-PCL, PGS-nonporous PCL and Bilayer PCL grafts (top lane). **b**, **d and f**, SEM images present the microstructures of 3min-PGS-PCL, PGS-nonporous PCL and Bilayer PCL grafts (top lane). **g**, **h and I**, These grafts were interposed into abdominal aorta in rats (middle). **j**, **k and l**, Gross appearance of grafts showed variant remodeling of these grafts through 14 days (Bottom Lane). **m**, Collagen membrane was wrapped around the 3min-PGS-PCL graft to block the perivascular adipose tissue. **n**, Silicon plugs were placed at the anastomosis sites to block the adventitia of adjacent vessels. **o and p**, Gross appearance of the grafts treated by two physical shields through 14 days. Black arrow: Collagen membrane. White arrows: Silicon plugs. G: Graft. N: Native artery.

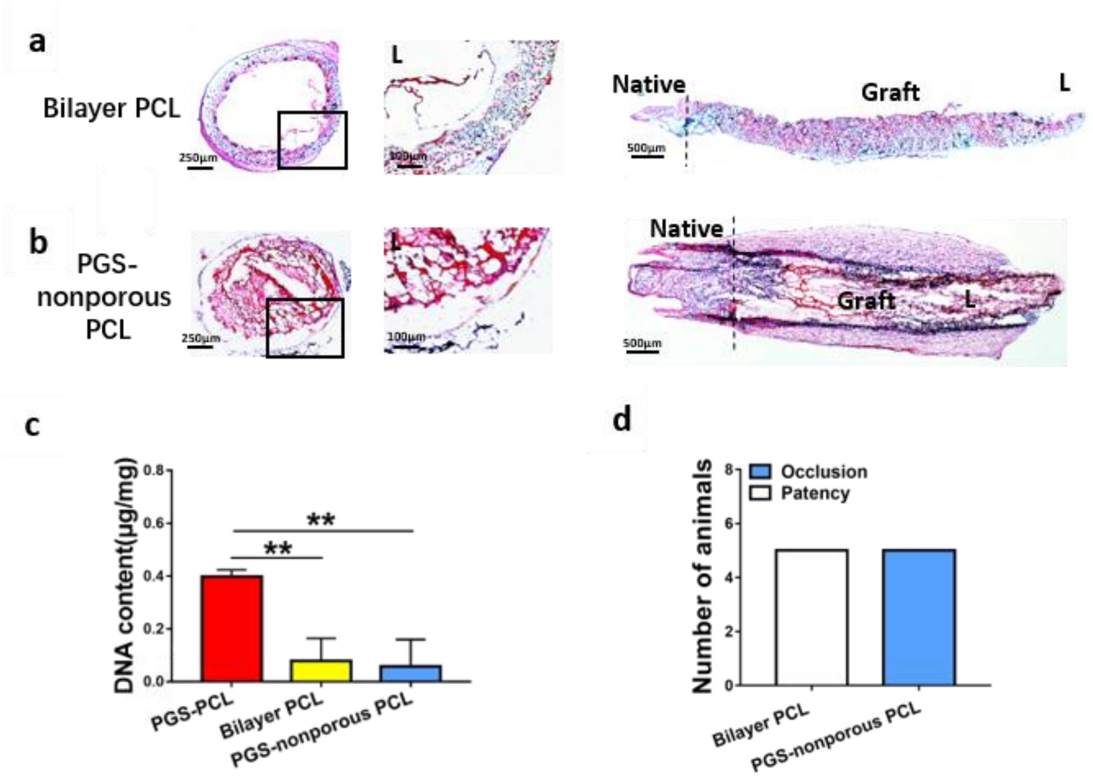

Figure S3. Tissue remodeling and cellularization of neoarteries under different blocking conditions. a and b, H&E staining of neoarteries in bilayer PCL and PGS-nonporous PCL grafts at 14 days post-implantation. L: Lumen. **c**, Comparison of DNA content (n=3 independent samples). **d**, Comparison of patency rate (n=5 independent samples). Data are represented as the mean ± SD for each group. For **c**, significance was determined by one-way ANOVA followed by Tukey's post hoc analysis. ******: p <0.01

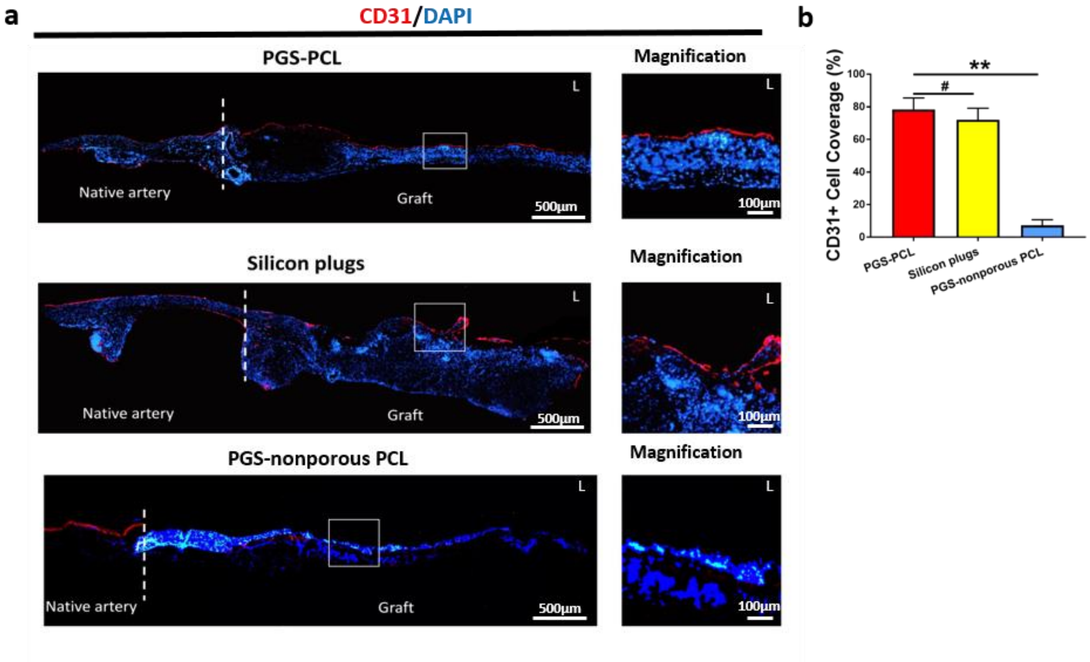

Figure S4. Endothelialization of grafts under different blocking conditions. a, Representative immunofluorescence images of PGS-PCL, silicon plus and PGS-nonporous PCL groups show endothelialization presented as CD31 immunostaining after 14 days' implantation. DAPI was used to counterstain the nuclei. **b**, Quantification of endothelial coverage by determine the percentage of CD31 positive cells of total length (n=3 independent samples). Data are represented as the mean ± SD for each group. For **b**, significance was determined by one-way ANOVA followed by Tukey's post hoc analysis. #: $p > 0.05$, **: $p < 0.01$.

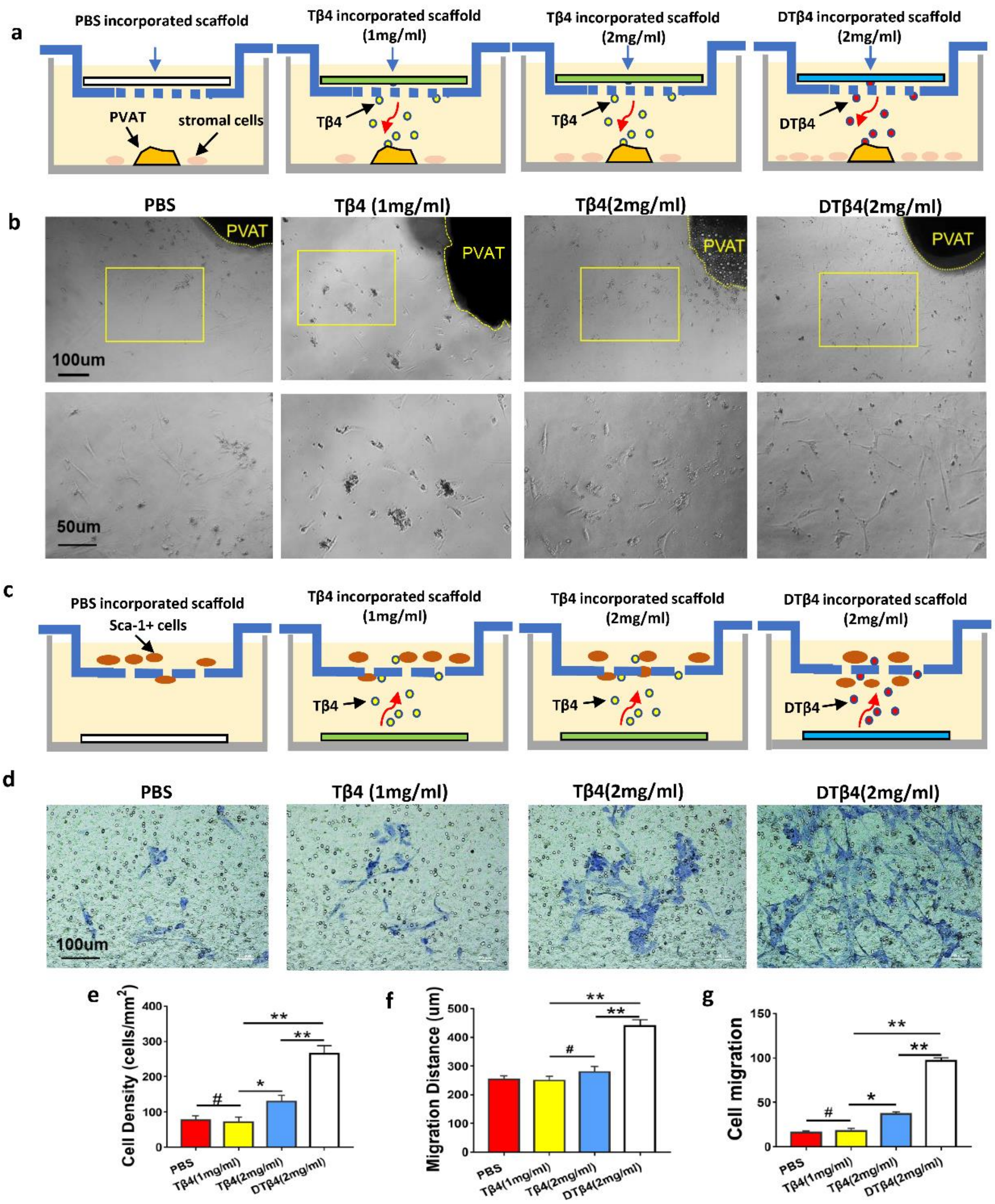

Figure S5. Examining biological activity of peptides released from the nanofibrous sheath. a, Schematic diagram of Trans-well system. **b,** Bright-field images reveal the outgrowth of stromal cells induced by peptides released from the nanofibrous sheath. **c,** Schematic diagram of trans-well migration assay. **d,** Images of migrated Sca-1+ cells induced by peptides released from the nanofibrous sheath. **e** and **f,** Quantitative analysis of cell outgrowth from PVAT stimulated by peptides released from the nanofibrous sheath (n=5 independent samples). **g,** Comparison of the number of migrating cells stimulated by peptides released from the nanofibrous sheath (n=5 independent samples). Data are represented as the

mean ± SD for each group. For **e-g**, significance was determined by one-way ANOVA followed by Tukey's post hoc analysis. #: $p > 0.05$, *: $p < 0.05$, **: $p < 0.01$

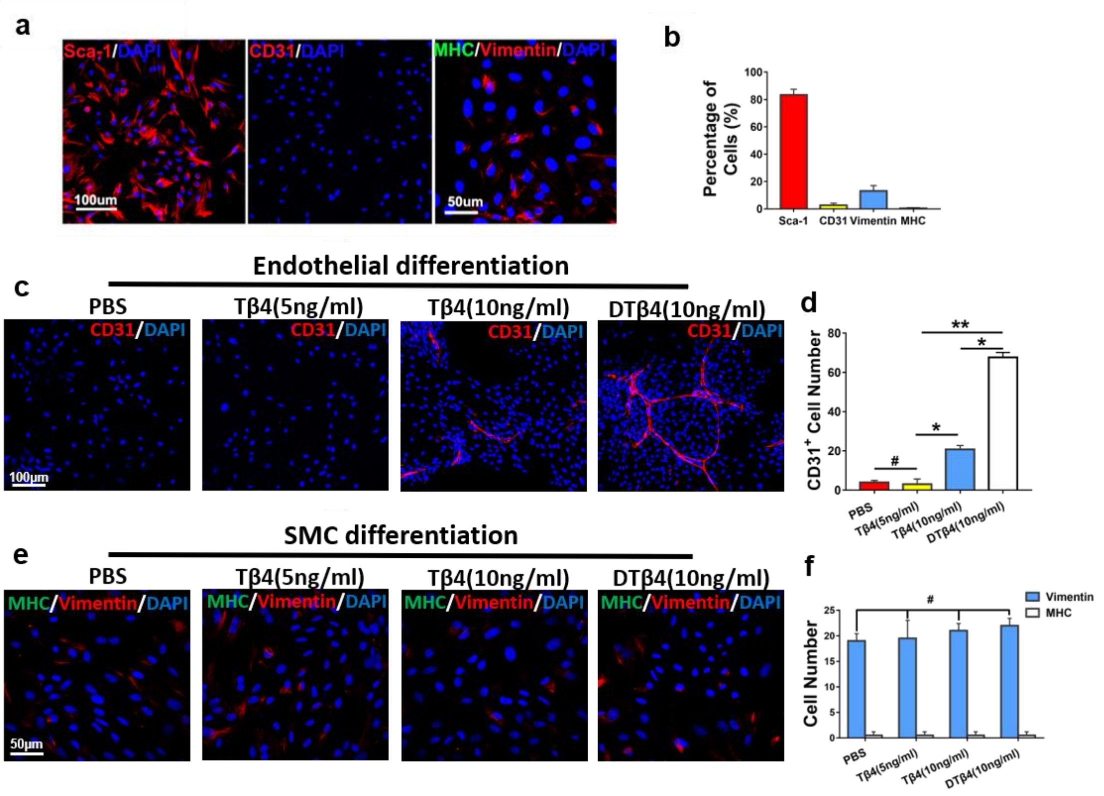

Figure S6. a, Identification of PVAT-derived cells by immunofluorescence staining. DAPI was used to counterstain the nuclei. **b,** Quantification of the percentage of Sca-1+, CD31+, Vimentin+ and MHC+ cells in the total number of PVAT-derived cells (n=5 independent samples). **c** and **e**, Representative immunofluorescence images show the endothelial differentiation (CD31+) and SMC differentiation (MHC+/vimentin-) of PVAT-derived cells stimulated by peptides released from the nanofibrous sheath. DAPI was used to counterstain the nuclei. **d** and **f**, Counting CD31+ cells and MHC+ /Vimentin- cells stimulated by peptides released from the nanofibrous sheath (n=5 independent samples). Data are represented as the mean ± SD for each group. For **d** and **f**, significance was determined by one-way ANOVA followed by Tukey's post hoc analysis. #: $p > 0.05$, *: $p < 0.05$, **: $p < 0.01$.

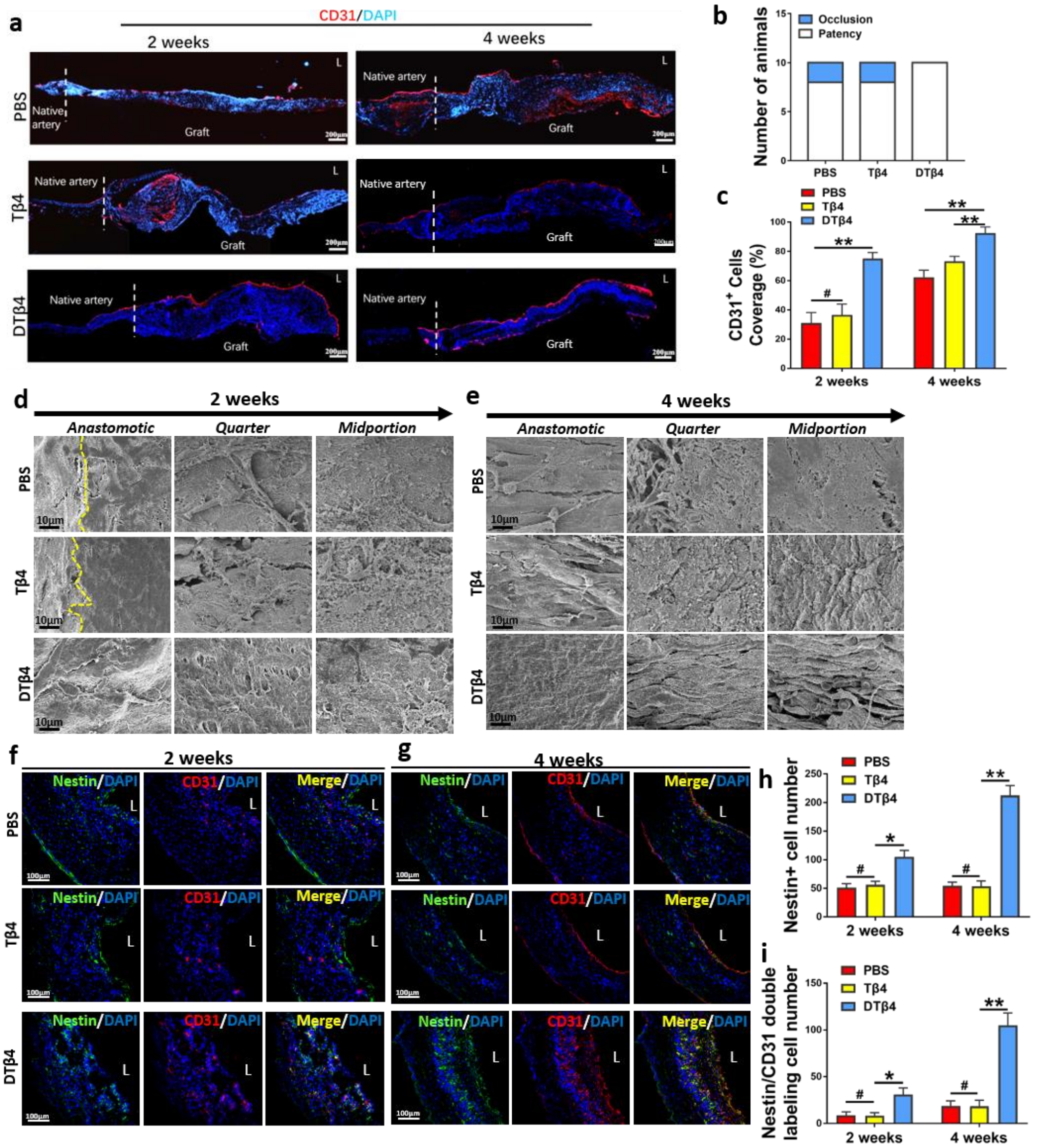

Figure S7. DTβ4 effectively increased endothelialization of the grafts. **a**, The representative images of CD31 immunofluorescence staining of longitudinal section. DAPI was used to counterstain the nuclei. The dotted line marks the anastomotic site. L: lumen. **b**, Comparison of patency rate among PBS, Tβ4 and DTβ4 groups (n=10 independent samples). **c**, Quantification of endothelial coverage by determining the percentage of CD31 positive region from total length (n=3 independent samples). **d and e**, SEM images show the endothelial coverage in lumen of PBS, Tβ4 and DTβ4 grafts at 2 weeks and 4 weeks post-implantation. The yellow dotted line represented the borders of native arteries and neoarteries. The borders of PBS and Tβ4 groups are clear, while the border of the DTβ4 group was covered by cells at two weeks post-implantation. **f and g**, Immunofluorescence staining images show the recruitment of neonatal ECs by using anti-Nestin and anti-CD31, colocalization was presented by double immunofluorescence staining with anti-Nestin (green)

and anti-CD31 (red). L: lumen. **h and i**, Quantification of the Nestin+ cells and Nestin+/CD31+ cells infiltrating into the grafts in PBS, Tβ4 and DTβ4 groups at 2 weeks and 4 weeks post-implantation (n=3 independent samples). Data are represented as the mean ± SD for each group. For **c**, **h** and **i**, significance was determined by one-way ANOVA followed by Tukey's post hoc analysis. #: $p > 0.05$, *****: $p < 0.05$, ******: $p < 0.01$.

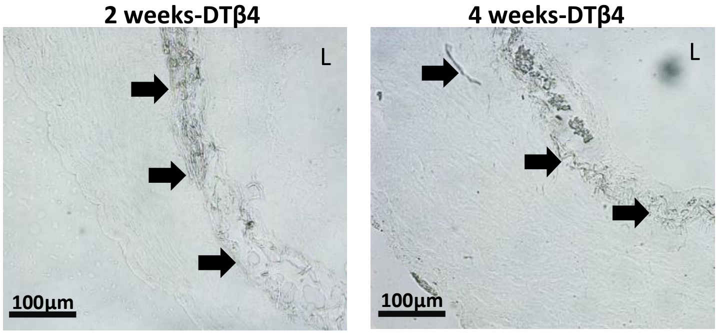

Figure S8. Bright-field images show the distribution of residual nanofibers in the neoarteries of DTβ4 group at 2 weeks and 4 weeks post-implantation respectively. Black arrows mark the residual nanofibers. L: lumen.

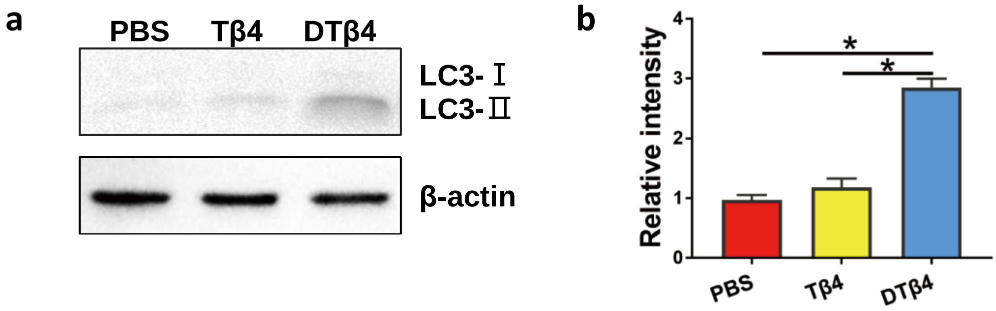

Figure S9. Western blotting reveals LC3 expression of the macrophages seeded on PBS, Tβ4 and DTβ4 loaded scaffolds at 7 days respectively (n=3 independent samples). Data are represented as the mean ± SD for each group. For **b**, significance was determined by one-way ANOVA followed by Tukey's post hoc analysis. *****: p <0.05.

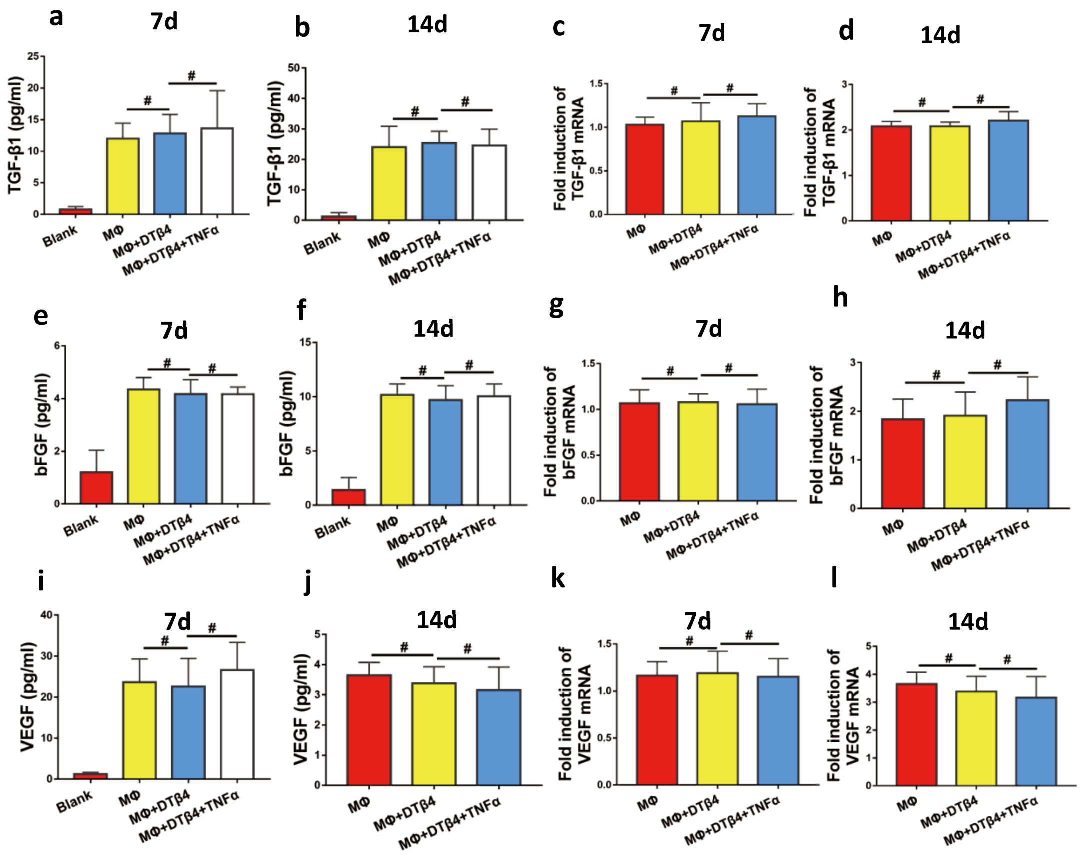

Figure S10. Quantitative analysis of angiogenic factors gene expression. (a, b, e, f, i, j) Quantification of TGF-β1, bFGF and VEGF secreted by macrophages in MΦ, MΦ+DTβ4 and MΦ+DTβ4+TNF-α groups at 7 and 14 days (n=3 independent samples). **(c, d, g, h, k, l)** Comparison of gene expression of TGF-β1, bFGF and VEGF of macrophages in MΦ, MΦ+DTβ4 and MΦ+DTβ4+TNF-α groups at 7 and 14 days (n=3 independent samples). mRNA expression of VEGF, TGF-beta1, bFGF were analyzed by RT-qPCR using the △△CT method. Significance was determined by one-way ANOVA followed by Tukey's post hoc analysis. #: $p > 0.05$.

f

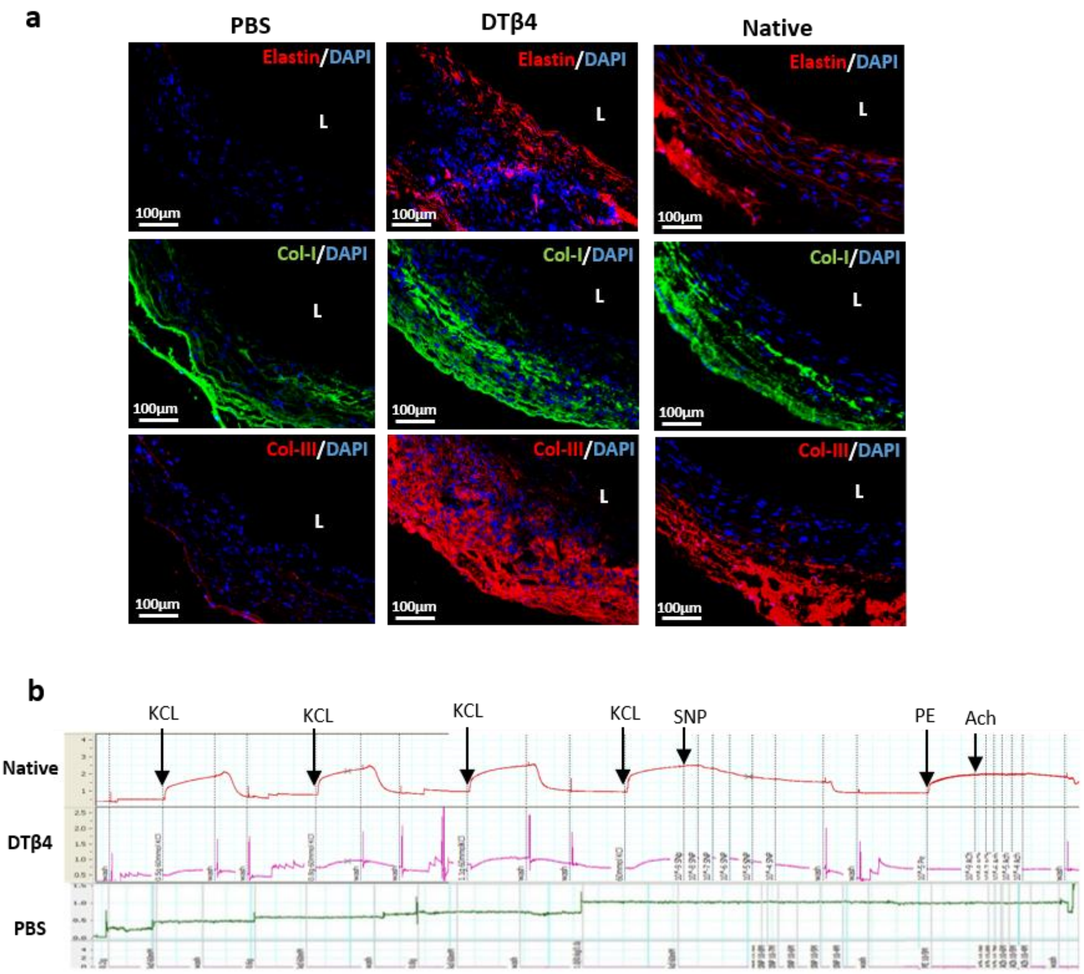

Figure S11. a, Representative immunofluorescent images of elastin (red), col-I (green) and col-III (red) in neoarteries from 3min-PBS and 3min-DTβ4 groups at 3 months post-implantation and native aorta. L: Lumen. **b**, Myographic examination of neoarteries from 3min-PBS and 3min-DTβ4 groups and native aorta under stimulation of chemical drugs such as potassium chloride (KCl), vascular smooth muscle cell specific activator SNP, selective α1-adrenergic receptor agonist phenylephrine (PE), and endothelial specific activator acetylcholine (Ach).

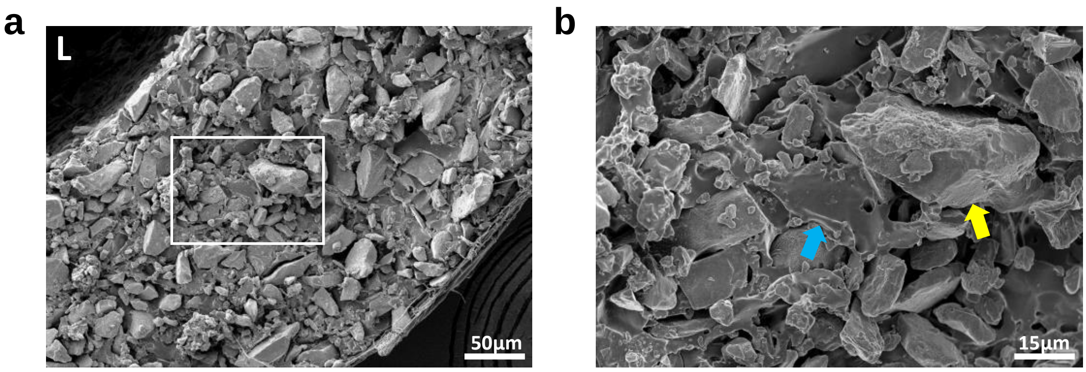

Figure S12. SEM images of PGS-sodium citrate tube. a, SEM shows that most of the pores in the PGS tube were occupied by sodium citrate. L: lumen. **b**, Higher magnification of **a**. Yellow arrow marked the sodium citrate and blue arrow marked the PGS.

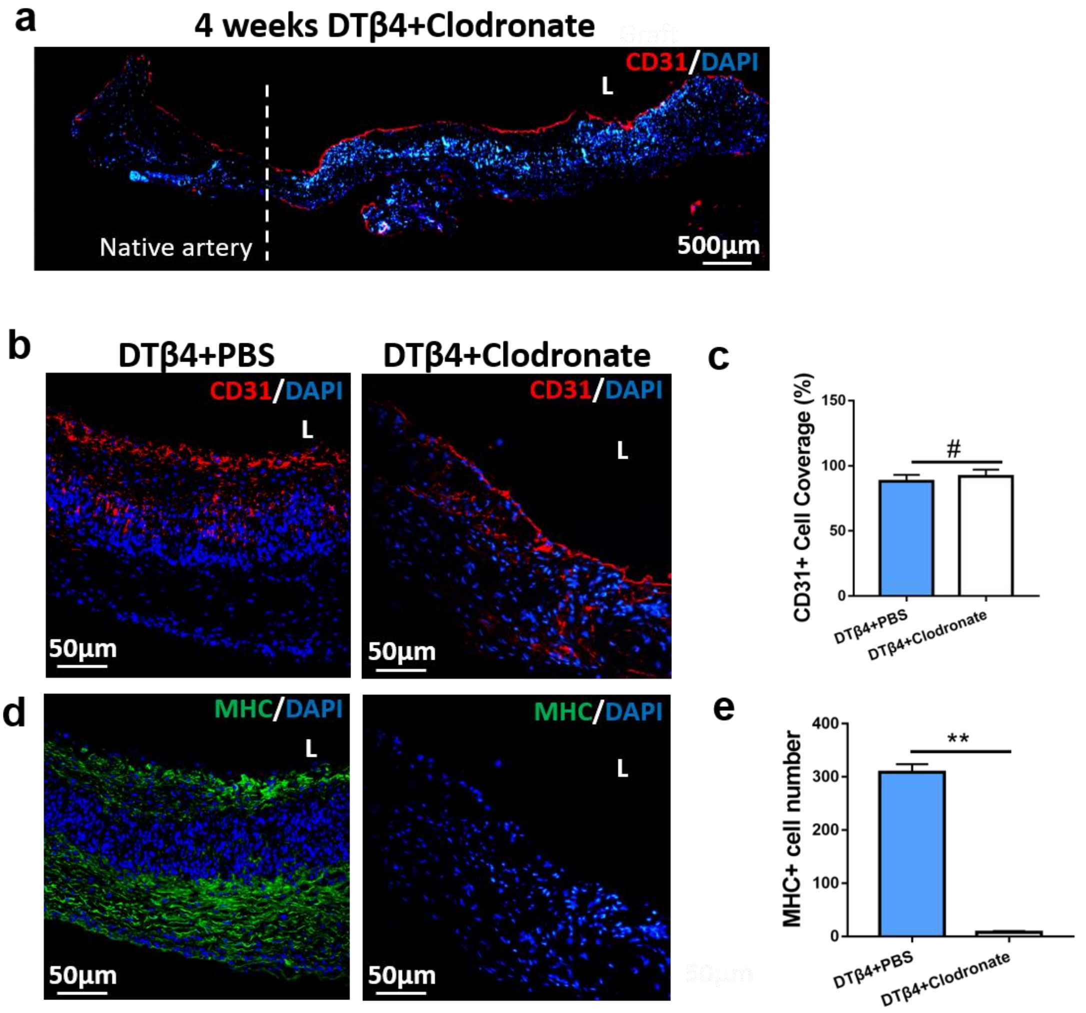

Figure S13. a, The representative images of CD31 immunofluorescence staining of longitudinal section for clodronate treated DTβ4 grafts at 4 weeks post-implantation. DAPI was used to counterstain the nuclei. The dotted line marks the anastomotic site. L: lumen. **b,** Immunofluorescence staining images show the recruitment of ECs by using anti-CD31. L: lumen. **c,** Quantification of endothelial coverage by determining the percentage of CD31 positive region from total length (n=3 independent samples). **d,** Representative immunofluorescent staining of MHC+ cells in neoarteries of PBS treated DTβ4 grafts and clodronate treated DTβ4 grafts at 4 weeks post-implantation. **e,** Quantification of MHC+ cells among DTβ4 + PBS group and DTβ4 + Clodronate group at 4 weeks post-implantation (n=3 independent samples). Data are represented as the mean ± SD for each group. For **c** and **e**, significance was determined by student t-test. #: $p > 0.05$, **: $p < 0.01$.

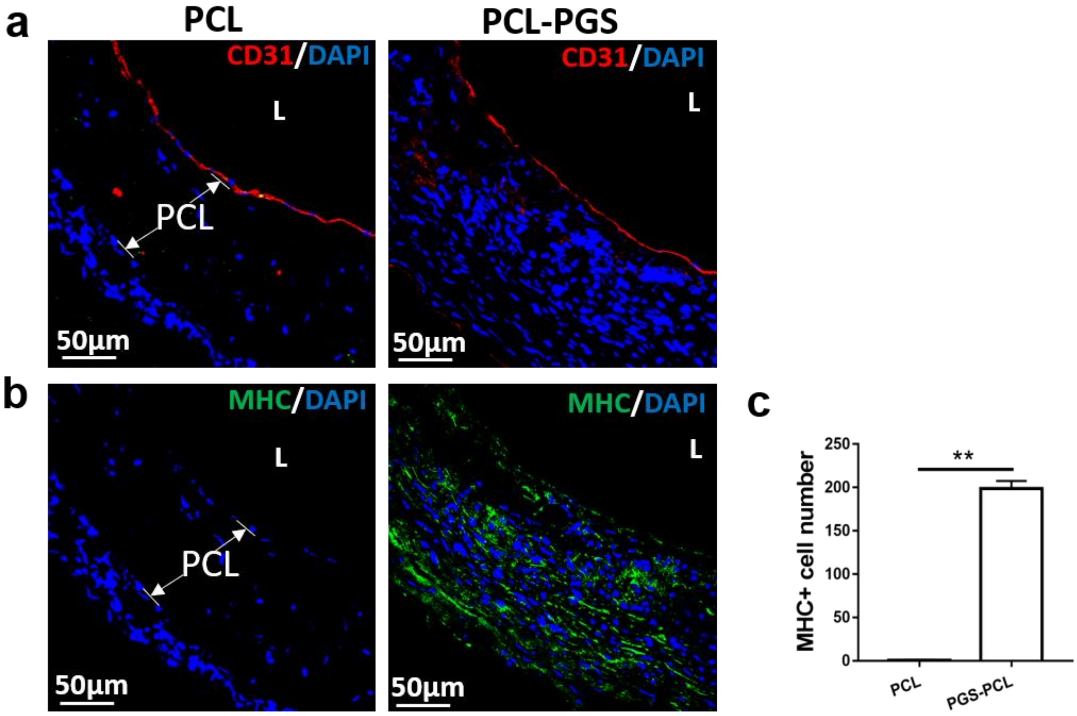

Figure S14. a, Immunofluorescence staining images show the recruitment of CD31+ ECs (red) in PCL grafts and PGS-PCL grafts at 4 weeks post-implantation. L: lumen. **b,** Representative immunofluorescent staining of MHC+ cells in neoarteries of PCL grafts and PGS-PCL grafts at 4 weeks post-implantation. **c,** Quantification of MHC+ cells among PCL group and PGS-PCL group at 4 weeks post-implantation (n=3 independent samples). Data are represented as the mean ± SD for each group. For **c**, significance was determined by student t-test. ******: $p < 0.01$.

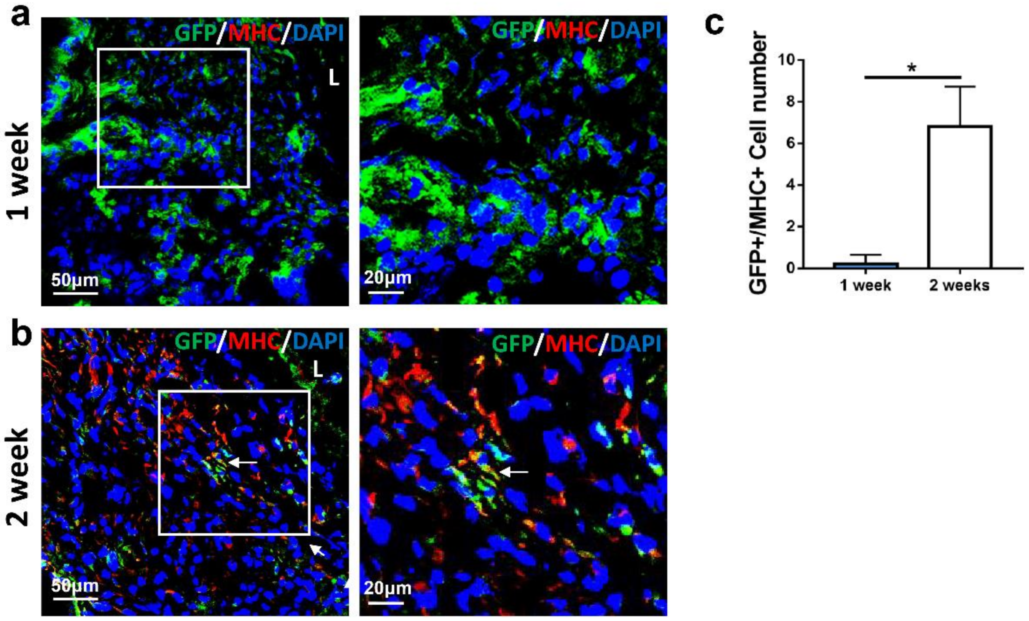

Figure S15. a and **b**, Immunofluorescence staining images show GFP+/MHC+ cells of GFP+ PVAT transplanted DTβ4 loaded grafts at 1 week and 2 weeks post-implantation. L: lumen. DAPI was used to counterstain the nuclei. **c,** Quantification of GFP+/MHC+ cells in GFP+ PVAT transplanted DTβ4 loaded grafts at 1 week and 2 weeks post-implantation. (n=3 independent samples). Data are represented as the mean ± SD for each group. For **c,** significance was determined by student t-test. *****: $p < 0.05$.

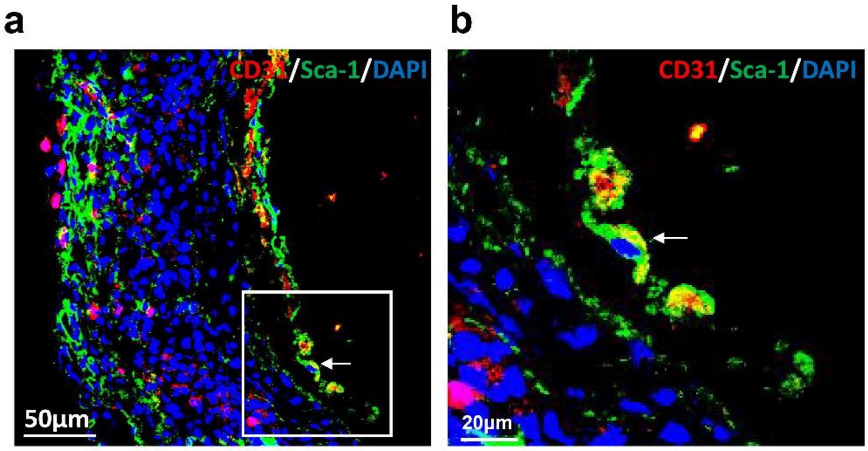

Figure S16. Immunofluorescence staining images show the recruitment of CD31+ ECs(red) and Sca-1+ cells (green) in cross-sections of DTβ4 loaded grafts at 2 weeks post-implantation. L: lumen. DAPI was used to counterstain the nuclei.
